# One-Pot Synthesis of Silver Nanoparticles Derived from Aqueous Leaf Extract of *Ageratum conyzoides* and Their Biological Efficacy

**DOI:** 10.3390/antibiotics12040688

**Published:** 2023-04-01

**Authors:** Deepak Paramasivam, Balamuralikrishnan Balasubramanian, Ramya Suresh, Jayanthi Kumaravelu, Manon Mani Vellingiri, Wen-Chao Liu, Arun Meyyazhagan, Amer M. Alanazi, Kannan R. R. Rengasamy, Vijaya Anand Arumugam

**Affiliations:** 1 Department of Life Science, Kristu Jayanti College (Autonomous), Affiliated to Bengaluru North University, Bengaluru 560077, Karnataka, India; 2 Department of Food Science and Biotechnology, College of Life Science, Sejong University, Seoul 05006, Republic of Korea; 3 PG and Research Department of Biotechnology, Dr. N.G.P. Arts and Science College, Autonomous and Affiliated to Bharathiar University, Coimbatore 641048, Tamil Nadu, India; 4 Department of Microbiology and Biotechnology, Bharth Institute of Higher Education and Research, Agharam Road Selaiyur, Chennai 600073, Tamil Nadu, India; 5 Department of Biotechnology, Rathnavel Subramaniam College of Arts and Science, Coimbatore 641402, Tamil Nadu, India; 6 College of Coastal Agricultural Sciences, Guangdong Ocean University, Zhanjiang 524088, China; 7 Department of Life Science, CHRIST (Deemed to be University), Bengaluru 560076, Karnataka, India; 8 Pharmaceutical Chemistry Department, College of Pharmacy, King Saud University, Riyadh 11451, Saudi Arabia; 9 Laboratory of Natural Products and Medicinal Chemistry (LNPMC), Department of Pharmacology, Saveetha Dental College, Saveetha Institute of Medical and Technical Sciences (SIMATS), Chennai 600077, Tamil Nadu, India; 10 Department of Human Genetics and Molecular Biology, Bharathiar University, Coimbatore 641046, Tamil Nadu, India

**Keywords:** *Ageratum conyzoides*, antibacterial, antidiabetic, silver nanoparticles, FE-SEM, HR-TEM

## Abstract

The main objective of the present research work is to assess the biological properties of the aqueous plant extract (ACAE) synthesised silver nanoparticles from the herbal plant *Ageratum conyzoides*, and their biological applications. The silver nanoparticle syntheses from Ageratum conyzoides (Ac-AgNPs) were optimised with different parameters, such as pH (2, 4, 6, 8 and 10) and varied silver nitrate concentration (1 mM and 5 mM). Based on the UV–vis spectroscopy analysis of the synthesised silver nanoparticles, the concentration of 5 mM with the pH at 8 was recorded as the peak reduction at 400 nm; and these conditions were optimized were used for further studies. The results of the FE-SEM analysis recorded the size ranges (~30–90 nm), and irregular spherical and triangular shapes of the AC-AgNPs were captured. The characterization reports of the HR-TEM investigation of AC-AgNPs were also in line with the FE-SEM studies. The antibacterial efficacies of AC-AgNPs have revealed the maximum zone of inhibition against *S. typhi* to be within 20 mm. The in vitro antiplasmodial activity of AC-AgNPs is shown to have an effective antiplasmodial property (IC_50_:17.65 μg/mL), whereas AgNO_3_ has shown a minimum level of IC_50_: value 68.03 μg/mL, and the Ac-AE showed >100 μg/mL at 24 h of parasitaemia suppression. The α-amylase inhibitory properties of AC-AgNPs have revealed a maximum inhibition similar to the control Acarbose (IC_50_: 10.87 μg/mL). The antioxidant activity of the AC-AgNPs have revealed a better property (87.86% ± 0.56, 85.95% ± 1.02 and 90.11 ± 0.29%) when compared with the Ac-AE and standard in all the three different tests, such as DPPH, FRAP and H_2_O_2_ scavenging assay, respectively. The current research work might be a baseline for the future drug expansion process in the area of nano-drug design, and its applications also has a lot of economic viability and is a safer method in synthesising or producing silver nanoparticles.

## 1. Introduction

The discovery of medicinal compounds from plant resources are of great interest in recent years. There are roughly 21,000 different plant varieties employed as alternative medicine [1]. However, 50,000 different species of medicinal plants are utilised as part of traditional medicine. Traditionally, medicinal plants were predominantly used by people from all over the world, as they are readily available and a chief source compared to modern medicine sold in pharmacies [2]. In 2012, it was estimated that about USD 2.2 billion revenue is generated every year only through the export of thousands of different species of medicinal plants. In this regard, there are a few guidelines amended and followed in different regions, but the WHO is also involved in the coordination of safe and rational usage of medicinal plants [3].

Malaria is a serious threat to humans, especially the people living on tropical and sub-tropical continents. The strategic report released from the WHO states that close to 2300 million people were at the risk of contracting a malarial infection. Malarial infection is a vector-borne disease caused and spread by the eukaryotic single-cellular organism or parasite called *Plasmodium falciparum* and the female *Anopheles stephensi* mosquito species, respectively. This deadly disease is one of the major causes of mortality and morbidity. Approximately 300 to 400 million new clinical cases are admitted every year for malarial treatment, with 1.5 to 2.7 million deaths recorded [4]. In addition to this, about one million deaths was reported for children who are under the age of 5 years, with the cause of death due to malaria alone or in combination with some other deadly disease. Annually, there are 2 to 3 million new cases of malarial infections were still reported in India, and it leads as the sole reason for death and other health complication faced by people in the country [5]. Improper maintenance of drainages and poor sanitation were the most favourable conditions, and the disease is endemic in the entire country. In addition to this, the malarial infections in the country have consumed huge investment amounts for drug production and labour costs for treatment. In contrast to this, the infective parasite is constantly developing its mechanism of resistance to antimalarial drugs. Hence, many developing countries, instead of doing research on antimalarial compounds, are searching for natural-based metabolites for the eradication of the mosquito vector, which is an ideal method to minimise the population of the vector [6].

Next to the most prevalent disease of malaria, diabetes mellitus is a life-long disorder that affects a large population across the globe. It is a disorder of carbohydrate metabolism which leads to various problems, including hyperglycaemia, insulin resistance, dyslipidaemia, and secretion of insufficient insulin. It is one of the most prevalent human health issues of both developing and developed countries. Frequent utilization of high-calorie-containing food stuffs, increased body weight, less physical activity and an inactive routine lifestyle are the major causes for the increase in diabetic cases [7]. The World Health Organisation (WHO), during its 2013 report, stated that about 90% of people with diabetes from all over the world were suffering with Type 2 Diabetes Mellitus (T2DM). More than 80% of the death rate in the world’s total population occurs due to the incidence of diabetes, in which India stands in second place. It has been estimated that about 366 million people from around the globe were expected to develop into diabetics by the year 2030, because of the lack of identification of effective diabetic medication/formulation that provides long-term glycaemic inhibition with fewer or no side effects. Perez-Gutierrez and Damian-Guzman have studied the inhibitory role of digestive enzymes in postprandial hyperglycaemia; their ingestion is an effective tool and the best method to control carbohydrate digestion [8]. According to a report released by the World Health Organization in the year 2013, diabetes alone will be the major and seventh primary cause of death by the year 2030 [1]. The currently available diabetic medications in the market, including acarbose, metformin, migllitol and voglibose, control carbohydrate digestion via inhibiting the effect exerted by the digestive metabolites of alpha amylase and alpha glucosidase. However, in addition to the effective formulation of the above-mentioned drugs, the frequent usage of such diabetic medications are also reported to have various side effects, including adverse gastrointestinal effects and liver and kidney failure [9].

During the 19th century, due to the advancements acquired in chemical analysis, studies have slowly replaced the concepts and preparation of medicine from natural plant resources. Moreover, many initiatives were taken, and awareness raised through conducting research on such medicinally important plants from the 20th century to the 21st, with special reference to anticancer and hepatoprotective drug analysis with plant resources. The major intention of the usage of medicinal plants in medicine preparation is to maintain good and hygienic health, and to administer for specific health condition in both modern and traditional medicine formulations. Traditional medicines and their analogues support more than 80 percent of the total world population. Regular consumption of medicines and food materials derived from natural resources, including natural health products and herbal food stuffs enriched with added health benefits, tend to be increased in developed countries. The farming of medicinal plants has many potential benefits, including the health benefits to the persons who consumes and economic benefits to the people who harvest and to the agents who process the products and commercialise them in society [10].

Recently, the field of nanotechnology has often been the focus of various researchers around the world due to the size, dimension and surface of nanomaterials. The synthesis of nanoparticles from various natural resources and chemical synthesis methods have presented a great opportunity in antibacterial studies, as they can penetrate the biological membrane system of the bacteria and are benign in their action [11,12]. Among the different raw materials used for nanoparticle synthesis, the aqueous extraction of different plant parts was reported to have better efficacy in various biological assays [6]. Moreover, nanoparticle formulation is applied in a variety of application-oriented research, including wastewater treatment, dye degradation, pharmaceutical agent formulation, and cosmetic industries and so on. Recent studies have reported that the formation of silver nanoparticles from different biological resources offers a wider spectrum of applications than the traditional techniques employed. As silver metal ions are noted for their high antimicrobial property, making different utensils and ornaments using silver has been practiced since ancient times. As the action of the nanoparticles is very high, it is being tested for various biological activities such as anticancer, antifungal, antiviral, antioxidant and antidiabetic [12,13]. Many research papers have reported nanoparticle synthesis using aerial plant parts, including *Ageratum conyzoides* and *Ageratum houstonianum* [6,13,14,15,16]. However, such studies failed to report the optimization and stabilization procedures of the green-synthesised nanoparticles. In our present study, the preparation of silver nanoparticles prepared with *Ageratum conyzoides* is optimised with two different parameters: pH (2, 4, 6, 8 and 10) and concentration of the nanoparticle precursor solution (1 mM and 5 mM). This is the significance of this study and its difference from earlier reports.

*Ageratum conyzoides* (billy-goat weed) is a simple and terrestrially invasive plant in India; it is native to tropical America, specifically Brazil. It is also a highly invasive weed species in most of regions around the world. *A. conyzoides* is a common tropical weed, which is most susceptible to spreading as it has entered even the subtropical and temperate zones, where this plant is also growing in the summer season. Different parts of these plant materials, due to antiseptic activity, were often used to cure many biological discomforts including arthrosis, headache, dyspnoea and common wounds. This plant is classified as a wide-spaced annual crop and under the permanent tree crops. This plant is found to be well-adapted to various kinds of habitat, including forest, grasslands, wastelands, clearings, wetlands, coastal dunes and roadsides and rapidly colonizes cultivated areas of both heavy and light soil nature. This plant has no specific photoperiod requirement. Hence, the selected plant of the study can be easily cultivated on a large scale for human consumption and for some medicinal purposes. This plant’s varieties are unable to self-pollinate and can only be pollinated by insects; they can produce over 40,000 seeds per plant. It can grow well in high-fertility soil along with dense populations when fertilizers are applied [9]. With this view, the focal aim of the present study was the synthesis, optimization and characterization of silver nanoparticles (AgNPs) from the aqueous extract of *A. conyzoides* (Ac-AE) and evaluation of their biological potential, with reference to the antioxidant, antibacterial and antiplasmodial efficacy and in vitro antidiabetic activity.

## 2. Results

### 2.1. Phytochemical Analysis of Ageratum conyzoides

**Figure 1:**
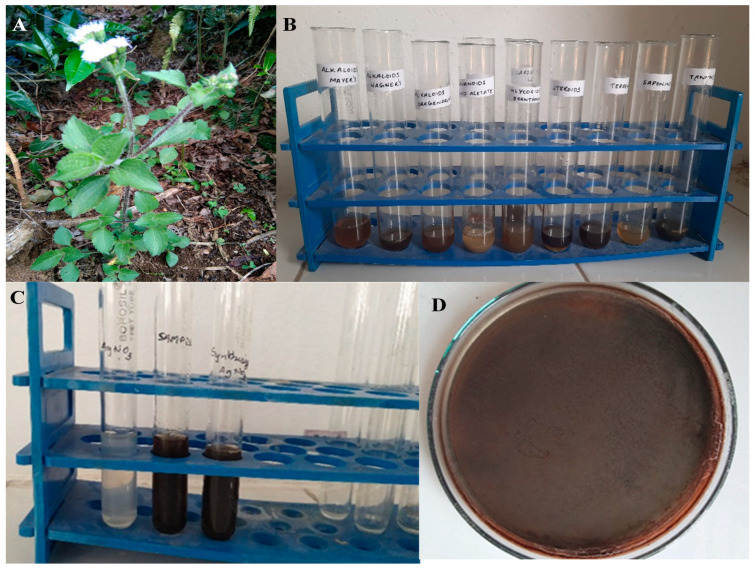
Phytochemical and AgNP synthesis from *Ageratum conyzoides*. (**A**) Plant part of *Ageratum conyzoides*; (**B**) Phytochemical analysis of Ac-AE; (**C**) Synthesis of Ac-AgNPs; and (**D**) Centrifuged Ac-AgNPs.

Phytochemical analyses of the *Ageratum conyzoides* aqueous leaf extract (AC-AE) revealed the presence of all the tested plant phytochemicals, which confirms its medicinal property (Figure 1). The phytochemical screening revealed the presence of alkaloids, steroids, saponins, tannins and cardiac glycosides, and the absence of flavonoids, glycosides, terpenoids and resins was recorded in the AC-AE (Table 1).

### 2.2. Silver Nanoparticle Synthesis

The AgNO_3_ dissolved in the aqueous solution was reduced to Ag ions through the reduction process carried out by the green-plant extract of *Ageratum* sp. The reaction started within 2 h of the incubation with silver nitrate (5 mM). This was confirmed through appearance of formation of a pale-brownish appearance in the nanoparticle solution (Figure 1C). It was synthesised at various pH of 2, 4, 7, 8 and 10. The synthesised silver nanoparticles at pH 8 were taken for further characterization studies. The UV-Visible spectrum is the preliminary characterization of silver nanoparticles, and it can be considered to identify the physical property of the plant-based nanoparticles. The Ac-AgNPs produced at 1 mm showed no effective results, as represented in the Supplementary Figures S1a–e and S2a–e). The synthesised Ac-AgNPs using the aqueous leaf extract at 1 mM concentration did not show any effective results, whereas the sample which was synthesised at 5 mM concentration showed effective results when compared to 1 mM. The AgNPs synthesised at 5 mM at various pH have many absorbance peaks at various pH and showed effective results. At pH 8, it is found that the absorbance is near 420 nm, so pH 8 was taken for further characterization studies of the silver nanoparticles. The bulk synthesis of the Ac-AgNPs was performed, and they were centrifuged and air-dried for further analysis (Figure 1D).

### 2.3. Characterization of Silver Nanoparticles

#### 2.3.1. X-ray Diffraction (XRD) Studies of AC-AgNPs

**Figure 2:**
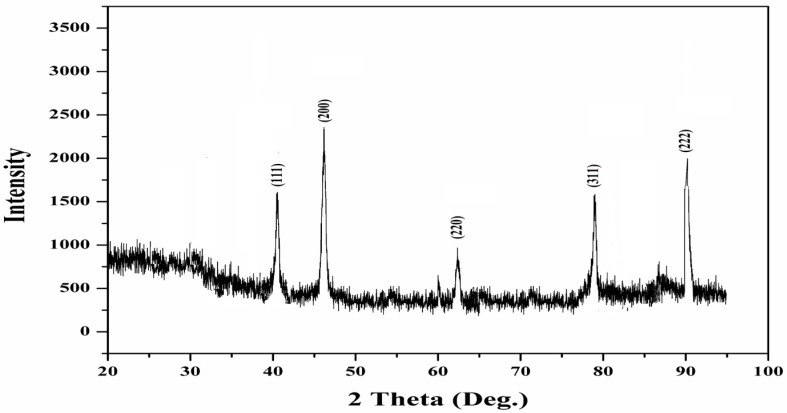
The X-ray diffraction (XRD) pattern of Ac-AgNPs.

The X-ray diffraction studies of the synthesised AgNPs showed the 2-theta values of 38.00°, 46.55°, 65.06° and 77.4°, corresponding to (1 1 1), (2 0 0), (2 2 0), (3 1 1) and (2 2 2), respectively (Figure 2).

The average crystalline size of synthesised Ac-AgNPs is ~39 nm, calculated using the Debye–Scherrer formula. Figure 2 depicts the strong and narrow diffraction peaks, indicating that the product is well crystalline. The XRD peaks at 38.00° and 46.55° are showing a similarity index to the Bragg’s reflections of cubic structure of metallic silver (JCPDS card no. 04-0784). The XRD peaks obtained for the AC-AgNPs are supporting the results of the FESEM analysis of the AC-AgNPs.

#### 2.3.2. FT-IR (Fourier Transform Infrared Spectroscopy) Analysis of Ac-AE and Ac-AgNPs

**Figure 3:**
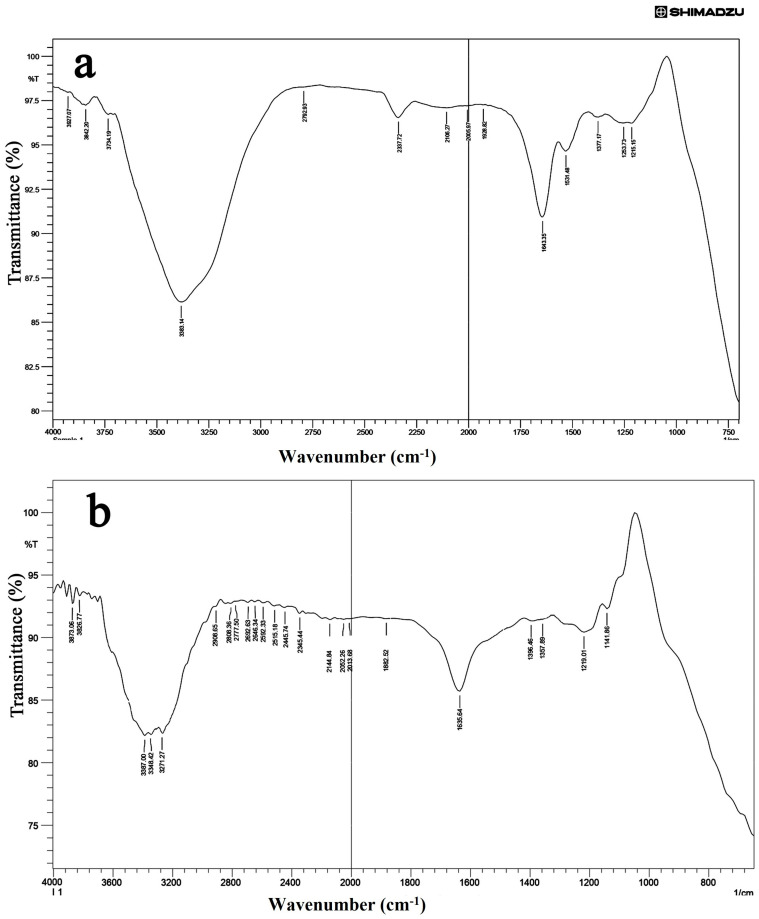
The FT-IR (Fourier Transform Infrared Spectroscopy) analysis of Ac-AE (**a**) and Ac-AgNPs (**b**).

The FT-IR analysis was carried out to understand the possible mechanism that leads to the synthesis of silver nanoparticles from the possible interactions between functional groups of the aqueous plant extract of *Ageratum conyzoides* and silver nitrate (Figure 3a,b). The spectral data were in the wavelength range of 4000–750 cm^−1^. The FTIR spectrum of Ac-AE showed peaks around 3927.07, 3842.20, 3734.19, 3383.14, 2792.93, 2337.72, 2106.27, 2005.97, 1928.82, 1643.35, 1531.48, 1377.17, 1253.73, and 1215.15 cm^−1^ (Figure 3a). The following functional groups were identified in the FT-IR analysis of Ac-AE: alcohols, phenols, aliphatic primary amine, aldehyde, allene, isothiocyanate, imine/oxime, alcohol (Table 2).

Similarly, the FT-IR analysis of silver nanoparticles synthesised from the aqueous extract of *Ageratum conyzoides* (Ac-AgNPs) showed the following peaks: 3873.06, 3826.77, 3387.00, 3348.42, 3271.27, 2906.65, 2806.36, 2777.50, 2692.63, 2345.44, 2013.68, 1635.64, 1219.01 and 1141.86 cm^−1^ (Figure 3b). The following functional groups were identified in the FTIR analysis of Ac-AgNPs: alcohols, phenols, aldehyde, carbon dioxide, imine/oxime, nitro compound, aromatic ester, vinyl ether, allene and isothiocyanate (Table 3).

### 2.4. Gas Chromatography and Mass Spectroscopy (GC-MS) Analysis of Ac-AE

**Figure 4:**
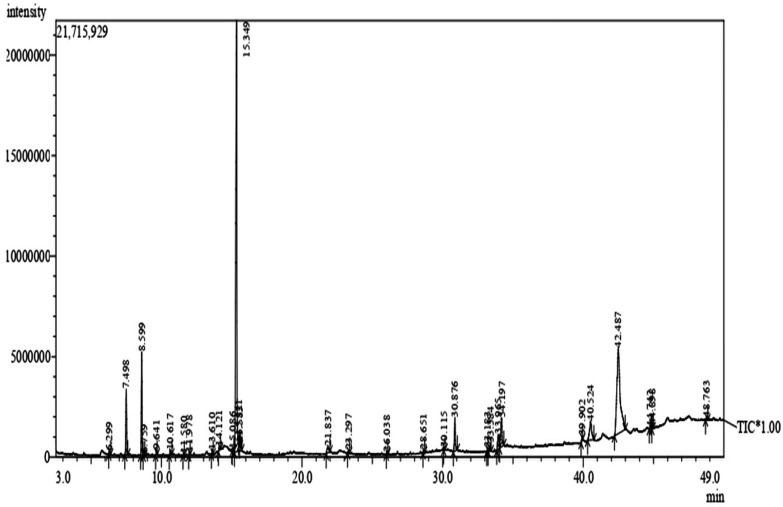
Gas chromatography and mass spectroscopy analysis of aqueous leaf extract of *Ageratum conyzoides* (Ac-AE).

The GC-MS analysis of the aqueous leaf extract revealed the presence of various biomolecules. The chromatogram of the Ac-AE showed many minor peaks in between, but also found some considerable major peaks with the following retention time: 7.498, 8.599, 15.349 and 48.763. The major peak recorded at the Rt of 15.349 was found to have a peak of phenol, 2-methyl-5-(1-methylethyl)- (Figure 4).

### 2.5. Field Emission Scanning Electron Microscopy (FE-SEM)

**Figure 5:**
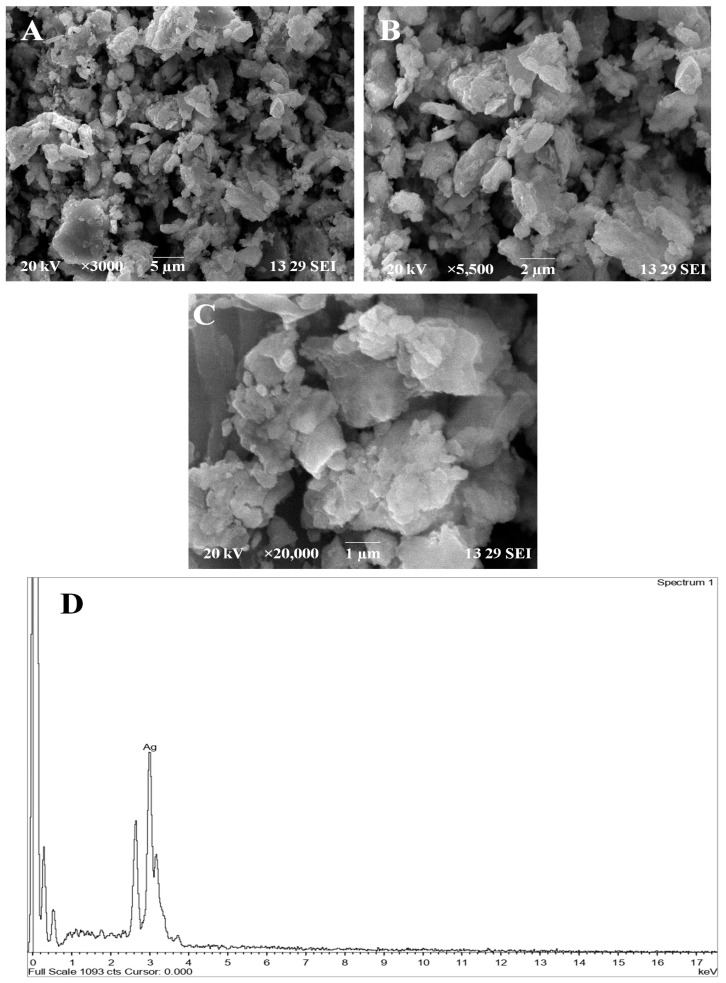
Field emission scanning electron microscopic (FE-SEM) images of AC-AgNPs (**A**) magnified at 3000×; (**B**) magnified at 5500×; and (**C**) magnified at 20,000×. (**D**) Energy dispersive X-ray spectroscopic analysis of Ac-AgNPs.

The FE-SEM analysis was used to determine the size and shape of synthesised silver nanoparticles. The SEM micrograph showed individual silver nanoparticle as well as the number of aggregates. Images at high resolution showed that the silver nanoparticles are well dispersed. The presence of secondary materials capping with the silver nanoparticles may be assigned to bio-organic compounds from the leaf extracts. The FE-SEM microscopic studies of the Ac-AgNPs clearly pointed out the size and morphology of the AgNPs (Figure 5). The micrographs of the Ac-AgNPs recorded the spherical shape of the nanoparticles, and the size ranges between ~30 and 90 nm (Figure 5A–C). The energy dispersive X-ray spectroscopy analysis of the Ac-AgNPs also confirmed the presence of silver ions in the sample (Figure 5D).

### 2.6. High-Resolution Transmission Electron Microscope (HR-TEM) Analysis of AC-AgNPs

**Figure 6:**
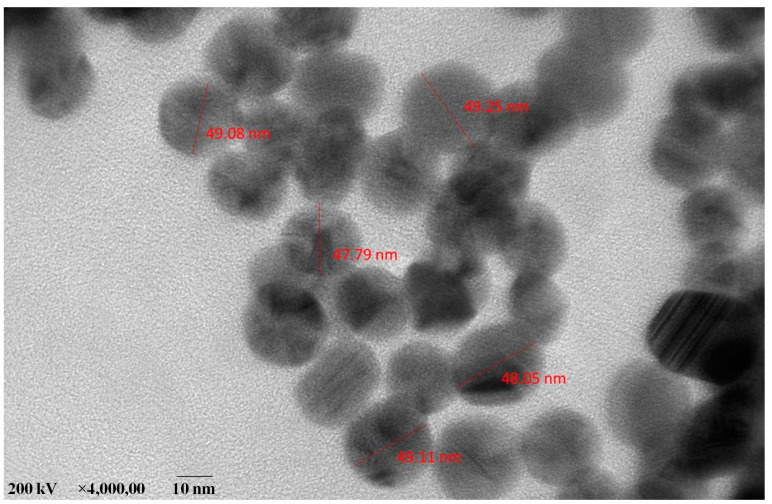
The high-resolution transmission electron microscope (HR-TEM) analysis of Ac-AgNPs.

The HR-TEM analysis of the AC-AgNPs in the present study explored the particle size and surface of the synthesised silver nanoparticles (Figure 6). The average size of the synthesised AgNPs present in the HR-TEM micrographic analysis of Ac-AgNPs closely resembles the results of the FE-SEM analysis.

### 2.7. Zeta Potential and Particle Size Distribution of AC-AgNPs

**Figure 7:**
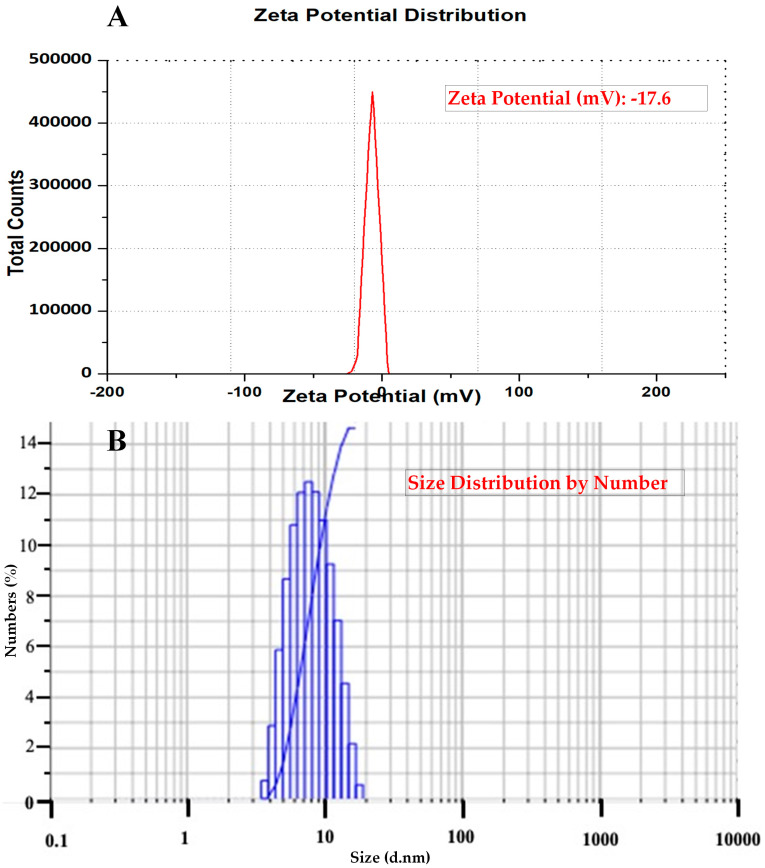
(**A**) Zeta potential values of AC−AgNPs and (**B**) particle size distribution of AC−AgNPs.

The zetasizer analysis of the Ac-AgNPs revealed the negative value of −17.6 mV, which confirms that the nanoparticles were in the colloidal state (Figure 7A). The particle size analysis showed that the Z-average of AC-AgNPs is 48 (d·nm), and PdI is 0.826 (Figure 7B).

### 2.8. Purity Analysis with High-Performance Liquid Chromatography (HPLC)

**Figure 8:**
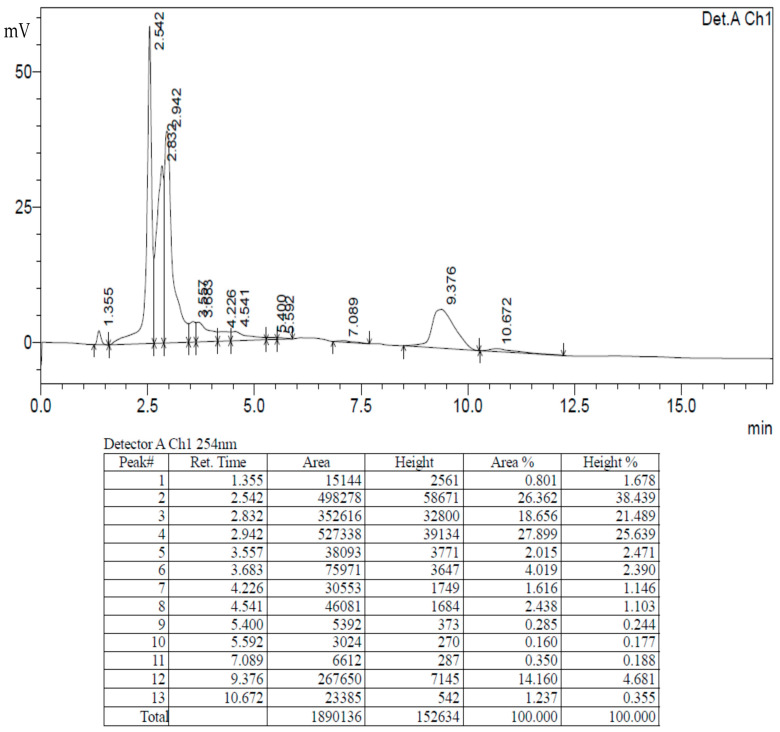
High-performance liquid chromatography analysis of aqueous extract of *Ageratum conyzoides* (AC-AE).

**Figure 9:**
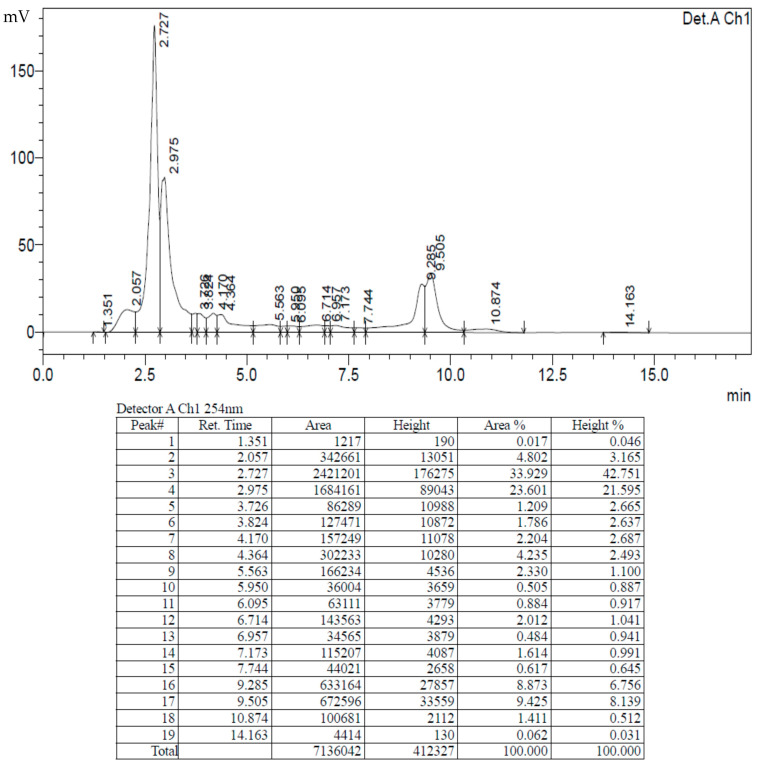
High-performance liquid chromatography analysis of Ac-AgNPs.

The HPLC analysis of the AC−AE and Ac−AgNPs revealed the presence of some common peaks in the chromatogram (Figure 8 and Figure 9).

### 2.9. Antibacterial Activity of Ac-AE and Ac-AgNPs

**Figure 10:**
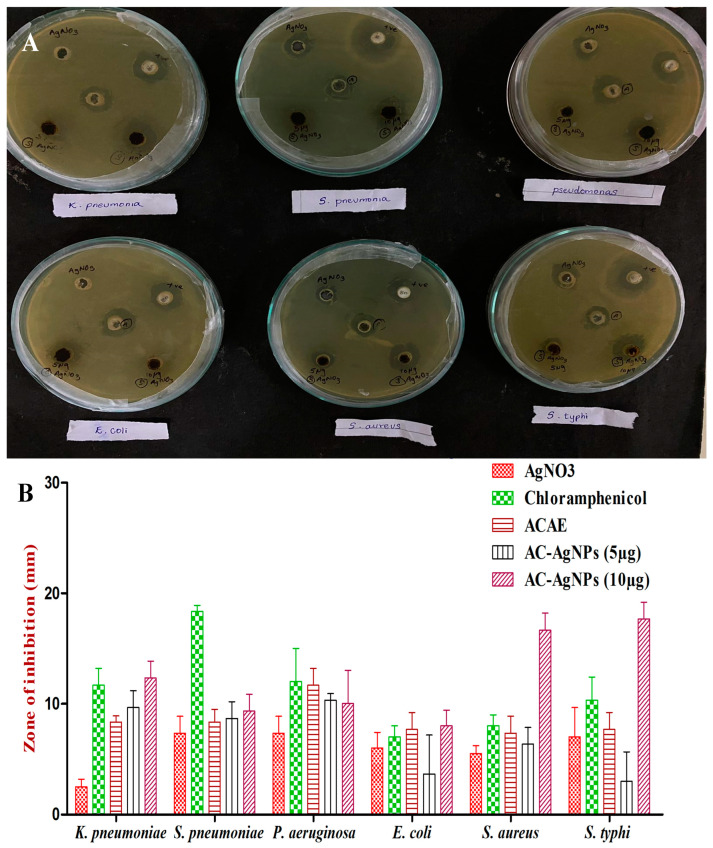
(**A**) Zone of inhibition recorded against selected bacterial pathogens and (**B**) antibacterial activity of AC-AE and AC-AgNPs against selected bacterial pathogens. Three replicates were performed.

The antibacterial activity of AC-AE and AC-AgNPs is shown in Figure 8. In the present study, AC-AgNPs of 5 µg and 10 µg were tested against six different bacterial species: *K. pneumonia*, *S. pneumonia*, *P. aeruginosa*, *E. coli*, *S. aureus* and *S. typhi*. *S. typhi* showed the highest zone of inhibition, 20 mm, for the AC-AgNPs (10 µg). *S. typhi* showed an 18 mm zone of inhibition for the AC-AgNPs (10 µg). *E. coli* was the bacterial species which showed the smallest zone of inhibition, 8 mm. *P. aeruginosa* and *S. pneumonia* inhibition zones were around 10 mm in the AC-AgNPs (10 µg). K. pneumonia showed a 13 mm zone of inhibition in AC-AgNPs (10 µg). Compared to AC-AgNPs (5 µg), AC-AgNPs (10 µg) showed higher zones of inhibition, indicating that the plant *Ageratum conyzoides* has greater antibacterial activity against the bacterial species (Figure 10A,B).

### 2.10. In Vitro Antioxidant Activity of Crude Plant Extracts

#### 2.10.1. DPPH Radical Scavenging Capacity

**Figure 11:**
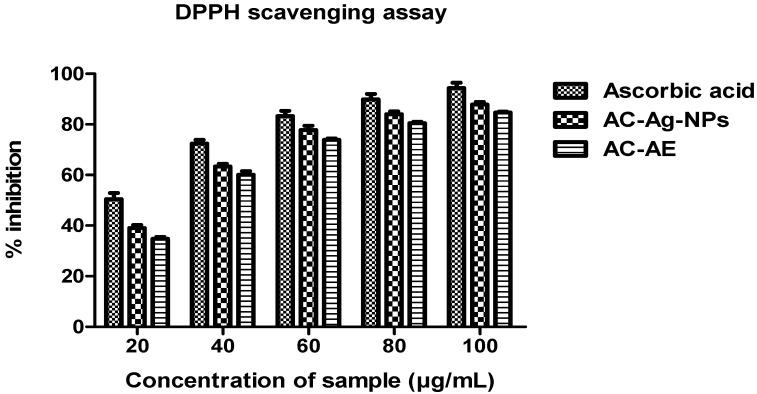
DPPH radical scavenging effect of *Ageratum conyzoides*. Three replicates were performed.

The DPPH radical scavenging capacity of AC-AE and AC-AgNPs was evaluated at different concentrations (20–100 µg/mL) of the sample, and the results are illustrated in Figure 11. The AC-AgNPs (87.86% ± 0.56) exhibited strong DPPH activity, followed by AC-AE (84.71% ± 0.17%). The positive control, ascorbic acid, exhibited a very high range of DPPH scavenging activity when compared to the crude extract (94.38% ± 1.21%). The IC_50_ values of the DPPH-scavenging properties of the AC-AE, AC-AgNPs and ascorbic acid were 64.51, 60.81 and 72.81 µg/mL, respectively.

#### 2.10.2. FRAP Radical Scavenging Assay

**Figure 12:**
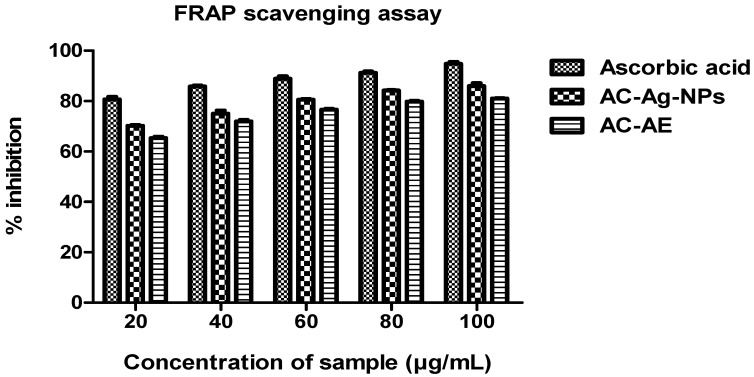
FRAP radical scavenging effect of *Ageratum conyzoides*. Three replicates were performed.

The ferric ion reducing capacity of the AC-AE and AC-AgNPs was evaluated at different concentrations (20–100 µg/mL), and the percentage of the scavenging property of each sample and control are illustrated in Figure 12. The AC-AgNPs (85.95% ± 1.02) exhibited strong FRAP activity, followed by the aqueous extract of *Ageratum conyzoides* (81.03% ± 0.15). The positive control, ascorbic acid, was found to have a very high range of FRAP scavenging activity compared to the aqueous crude extract (94.79% ± 0.74).

#### 2.10.3. H_2_O_2_ Radical Scavenging Assay

**Figure 13:**
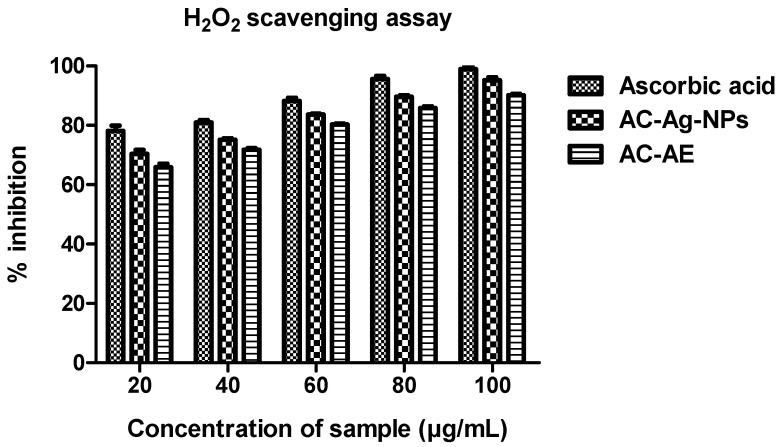
H_2_O_2_ radical scavenging activity of *Ageratum conyzoides*. Three replicates were performed.

The antioxidant activity of methanol extracts of the plant-based silver nanoparticles and the aqueous extract on hydrogen peroxide is shown in Figure 13 and compared with the standards. The extracts of *Ageratum conyzoides* and its nanoparticles were capable of scavenging hydrogen peroxide in an amount-dependent manner. Here, 100 µg/mL of methanol extracts of *Ageratum conyzoides* (Ac-AE) exhibited 95.7 ± 0.59% scavenging activity on hydrogen peroxide, followed by the nanoparticles of *Ageratum conyzoides* (AC-AgNPs) at 90.11 ± 0.29%. On the other hand, using the same amounts, ascorbic acid exhibited 98.93± 0.47% hydrogen peroxide scavenging activity.

### 2.11. In Vitro Antiplasmodial Activity of Ac-AE and Ac-AgNPs

**Figure 14:**
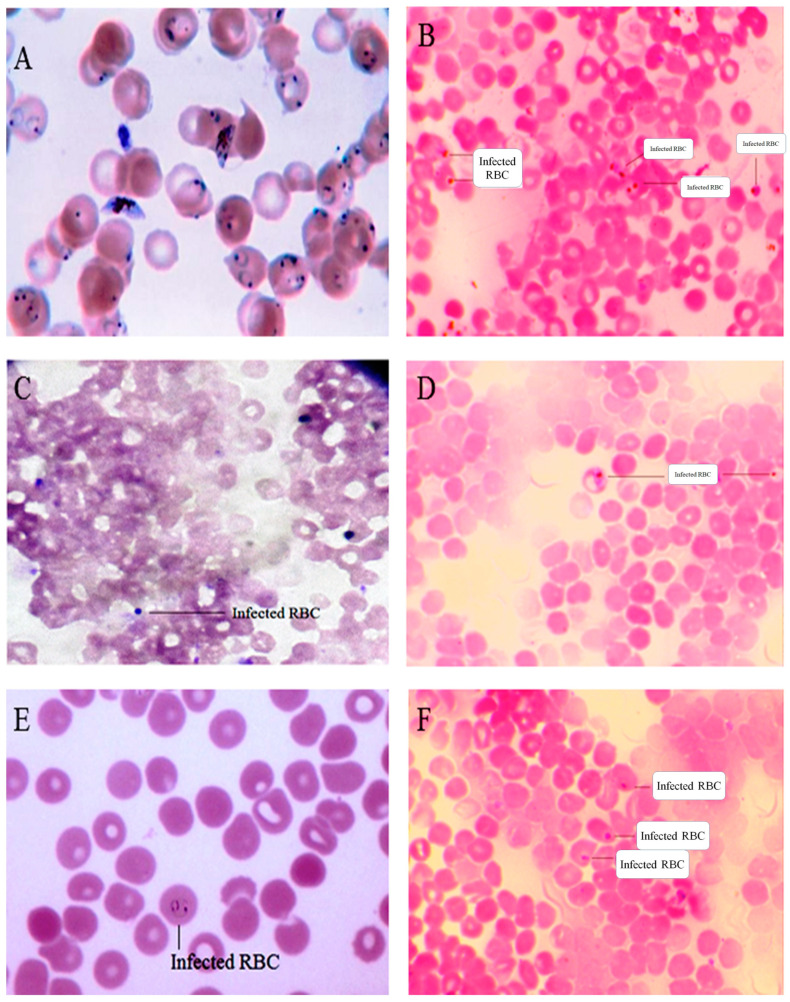
In vitro antiplasmodial activity of *Ageratum conyzoides* treated with (**A**). Negative control, (**B**). AC-AE, (**C**). AC-AgNPs, (**D**). AgNO_3_, (**E**). Chloroquine and (**F**). Artemether. Three replicates were performed.

The IC_50_ values of Ac-AE, AgNO_3_ and Ac-AgNPs against *P. falciparum* strains at 24 and 48 h of parasitaemia suppression are listed in Table 4. The antiplasmodial activity of AgNO_3_ had a minimum level of IC_50_ value 68.03 μg/mL, and the Ac-AgNPs and Ac-AE had 17.65 and >100 μg/mL, respectively, at 24 h of parasitaemia suppression. The IC_50_ value of Ac-AgNPs is comparable to the positive control, chloroquine (17.65 μg/mL). The IC_50_ value of Ac-AE, Ac-AgNPs and AgNO_3_ at 48 h of inhibition was 50.59, 10.87 and 40.90 μg/mL, respectively. The IC_50_ value at 48 h of inhibition of positive control, chloroquine was 12.5 μg/mL, which was less than Ac-AgNPs (10.87 μg/mL). In addition, artemether had a maximum level of IC_50_ value of 7.96 μg/mL. The microscopic observation of antiplasmodial treatment with Ac-AE, AgNO_3_ and Ac-AgNPs against *P. falciparum* is presented in Figure 14.

### 2.12. In Vitro Antidiabetic Activity of Ageratum conyzoides

**Figure 15:**
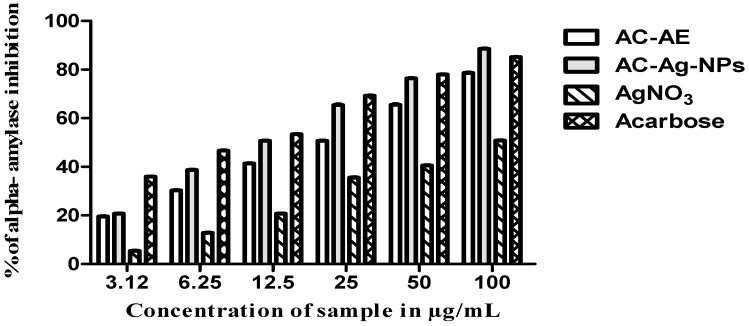
Graphical representation of α-amylase inhibition. Three replicates were performed.

The inhibitory activities against digestive enzymes (α-amylase) of acarbose (a commercial inhibitor of α-amylase) and the samples are equivalent (Ac-AE, Ac-AgNPs and AgNO_3_) are presented in Table 5 and Figure 15. The inhibitory capacities of samples against α-amylase (applied at concentration of 1.0 mg/mL) had a minimum IC_50_ (AC-AgNPs: 21.52 μg/mL), and moderate IC_50_ values (AC-AE: 37.02 and AgNO_3_: 85.46 μg/mL) were found.

## 3. Discussion

The earlier research work carried out by Puro et al. [17] reported that the phytochemicals present in the aqueous leaf extract of *Ageratum conyzoides* highly influence the antimicrobial activity, which is in line with the present study. Many research studies have suggested that the plant variety of *Ageratum* species is found to have excellent biological properties, as it is reported to have various phytochemicals, such as phenols, bromophenols, polysaccharides, tannins, flavonoids, phenolic acids and carotenoids [13,14,15,16,18,19]. The extract prepared form this plant material has been reported to have better activity in the following categories, including antibacterial [13,14], photocatalytic [15], antioxidant [16], antidiarrheal and antiplasmodial activities, anti-inflammatory, antipneumonial, antiasthma and antigastric [9]. The visual colour change in the aqueous leaf extract from brownish to pale brown confirms the initial synthesis process of silver nanoparticles as reported by [20]. The functional groups found in the plant extract through the FTIR report were also present in the silver nanoparticles, with the addition of few new peaks, confirms the structural changes that are involved in the reduction of silver nitrate to silver ions [21]. The morphology of the silver nanoparticles was predominantly spherical and aggregated into large irregular structures with no well-defined morphology [22]. The average size of the synthesised silver nanoparticles present in the HR-TEM micrographic analysis of Ac-AgNPs closely resembles the results of the FE-SEM analysis. Similar reports have been previously made by Singh et al. [23].

The chromatograms of the Ac-AE have shown many minor peaks in between, but also found some considerable major peaks with the following retention times: 7.498, 8.599, 15.349 and 48. 763. The presence of such peaks in the aqueous extract of the Ac-AE has influenced the formation of AgNPs in the present study. Similar findings were earlier reported by Bajpai et al. [24], who have identified 25 compounds using GCMS.

Over the last decades, several studies have reported on the antibacterial activity of terrestrial plant extracts [25]. The antimicrobial activity of plant extracts may be influenced by some factors, such as the habitat and the season of collection, different growth stages of the plant, experimental methods adopted, plant species, extraction methods followed, testing methodology, place of sample collection, and the plant regions and solvent used for extraction. Although a variety of solvents have been employed in screening plants for antimicrobial activity, it is still uncertain what kind of solvent is the most effective and suitable for the extraction of effective metabolites of plants [24].

Natural antioxidants are not limited to terrestrial sources, and reports have revealed that plants are also rich sources of natural antioxidant compounds. Phenolic compounds are commonly found in plants, including shrubs, and they have been reported to have a wide range of biological activities, including antioxidant properties [26]. Several studies have demonstrated a highly significant correlation between the phenolic content and the antioxidant activity in plant extracts. The phenolic compounds produced by the plant extracts significantly contribute to their antioxidant capacity when compared to other natural resources. The development of parasitic resistance to frontline antimalarial drugs such as Chloroquine and antifolates has underscored the importance of developing new drugs and drug targets to treat the disease. The continuous appearance of drug-resistant *P. falciparum* strains has made the chemotherapeutic management of malaria increasingly problematic in virtually all malarial regions of the world [27]. Ravikumar et al. [20] have reported that the different plant extracts possess various biological properties, such as antibacterial, antifungal, antiviral, anti-inflammatory, cytotoxic, nematicidal, antifeedant, larvicidal, phytotoxic, anticoagulant and spermicidal activities.

Many natural resources have been noted for their antidiabetic properties in Ayurveda for the treatment of diabetes. However, such resources have not gained much importance as medicines due to the lack of sustained scientific evidence [27]. Grover et al. [28] reported that the search for new pharmacologically active agents from natural sources can lead to the discovery of potent and specific inhibitors for α-amylase.

## 4. Materials and Methods

### 4.1. Plant Sample Collection

Fresh plant specimens of *Ageratum conyzoides* were collected from the local area of Coonoor, The Nilgiris, India. Then the plant samples were brought to the laboratory for further studies. Major studies of *Ageratum conyzoides* were performed in the laboratory located in Dr. NGP Arts and Science College, Coimbatore, Tamil Nadu, India; the antiplasmodial and antidiabetic studies and statistical analysis were performed by our research collaboration. The collected fresh plant samples were identified and authenticated by Dr. M.U. Sharief, Botanical Survey of India, Tamil Nadu Agricultural University, Coimbatore, Tamil Nadu, India. The authentication number is BSI/SRC/5/23/2022/87/Tech.

### 4.2. Aqueous Leaf Extract Preparation

The freshly collected healthy leaf materials of *Ageratum conyzoides* (Figure 1a) were processed by washing numerous times under running pipe-water to eliminate the associated biota and unwanted dust matters, followed by shading for 20 days under shade conditions. Then, the leaves were made into a fine powder using an electric mixer grinder. About ten grams of the powdered plant sample was mixed with a hundred millilitres of deionised distilled water and then kept in a shaker for 24 h. Then, the sample could be filtered using sterile muslin cloth [29].

### 4.3. Phytochemical Analysis of Ageratum conyzoides

The phytochemical analysis of the aqueous plant extract was carried out for qualitative estimation in the sample by using standard protocols. The following phytochemical analyses were performed to identify the occurrence of plant compounds, such as glycosides, steroids, flavonoids, alkaloids, terpenoids, saponins, tannins, acidic compounds and resins by adopting the standard protocol [30].

### 4.4. Nanoparticle Synthesis

The process of the silver nanoparticle synthesis was initiated by adding twenty millilitres of a fresh extracted aqueous leaf sample of *Ageratum conyzoides* (ACAE) with nanoparticle precursor solution. The nanoparticle synthesis procedure was optimised initially with 1 mM of silver nitrate solution at various pH (2, 4, 6, 8 and 10), mixed with the freshly prepared aqueous plant extracts. Based on the peak detection from the UV–vis spectrophotometer, the concentration of the silver nitrate was increased to 5 mM, and the synthesis procedure was performed at various pH (2, 4, 6, 8 and 10), with nanoparticles prepared and reduced from aqueous plant extract of AC-AE. The concentration AgNO_3_ solution and the aqueous plant sample were at a ratio of 1:9. Based on the band formation at 420 nm with the UV–vis spectrophotometry study, the nanoparticle solution prepared at 5 mM concentration along with the pH parameter of 8 was confirmed as optimal. Then, the silver nanoparticles (AgNPs) produced by the aqueous leaf extract of *A. conyzoides* (ACAE) were centrifuged at 15,000 rpm for 20 min (Cooling Microcentrifuge, REMI C-24 PLUS; Remi Elektrotechnik Limited Pvt. Ltd., Mumbai, India). The upper layer of the centrifuge tube after the centrifugation was discarded, and the so-called sediment pellets were parched, collected and air-dried. Then, the nanoparticle powder was collected in sterile Eppendorf tubes and stored for further characterization and biological applications [31].

### 4.5. Nanoparticle Characterization

#### 4.5.1. UV–Vis Spectroscopy Analysis

The changes observed in the sample colour concentration, from tea-green to brown, were a key indicator for the formation of nanoparticles in the solution. The biological formation of AgNPs was confirmed via the reduction of the AgNO_3_ through the metabolites of *Ageratum conyzoides* in the solution, tracked through checking the reading periodically using UV–vis spectroscopy at 420 nm [32].

#### 4.5.2. X-ray Diffraction Studies of AC-AgNPs

The synthesised and purified AgNPs were characterised using XRD analysis (X-ray diffractometer model: D8, Make: Brucker, Bremen, Germany) [32].

#### 4.5.3. Functional Group Analysis of AC-AE and AC-AgNPs

The functional group analysis of the plant sample and the synthesised silver nanoparticles was performed with FT-IR (Fourier Transform Infrared Spectroscopy) and noted between the arrays of 4000–400 cm^−1^ [33].

### 4.6. Gas Chromatography and Mass Spectroscopy Analysis of AC-AE

The bioactive metabolites present in the freshly prepared raw-aqueous extract of *Ageratum conyzoides* were analysed and recorded with the GC-MS method. The analysis of the bioactive compounds began with the injection of highly filtered 0.001 mL of the plant extract in the injection port (Perkin Elmer, Waltham, MA, USA). The helium gas (inert gas) was used as a mobile phase for bioactive compound separation. The peak identification and the molecular formula were compared with the spectra present in the Wiley library [34].

### 4.7. FE-SEM Analysis

The exact size and the structure of the synthesised Ac-AgNPs were examined through morphology analysis studies carried out with FESEM. The samples were processed after 24 h of incubation. The slides were first coated with the sample, and then thin layers of the platinum were applied to the sample, to become more conductive at the voltage of 20 kV [35].

### 4.8. HR-TEM Analysis of AC-AgNPs

The HR-TEM analysis of the particle studies revealed the measurements of particle size, distribution, morphology and grain size. To explore the nano-efficiency of the AC-AgNPs, the particles were subjected to HR-TEM microscopy studies (ESD; JEOL JEM 2100, Chicago, IL, USA) [36].

### 4.9. Particle Size Analysis of AC-AgNPs

The nanoparticle characteristics in the liquid solution were determined through zetasizer studies of AC-AgNPs, recorded with the zetasizer coupled with particle distribution analyser (Zeta-sizer, Malvern Instruments Ltd., Worcester, Worcestershire, UK) [36].

### 4.10. HPLC Analysis of AC-AE

To assess the purity of the plant sample, HPLC chromatographic examination was executed. The standard methodology of Anjum et al. [34] was adopted to compare the metabolite profile of the aqueous extract of *Ageratum conyzoides* and its nanoparticles.

#### 4.10.1. Preparation of Mobile Phase

##### Polar Extracts

The mobile phase of methanol:water (50:50) is used and exactly 1 mg/mL of the plant extract filtered through a syringe filter was completely mixed with 1000 microlitres of 100% HPLC grade methanol for the preparation of the 1 mg/mL solution. For the HPLC analysis, the plant samples were processed through the Shimadzu LC solution 20 AD, Japan. The instrument was coupled with a C18 column combined with a UV detector. The elution flow rate was initially assigned as 1 mL/min of the mobile phase.

### 4.11. Antibacterial Activity of Ac-AE and Ac-AgNPs

The antibacterial study was performed using the synthesised AgNPs against 6 different bacteria (*Pesudomonas aeruginosa*, *Klebsiella pneumoniae*, *Salmonella typhi*, *Escherichia coli*, *Staphylococcus aureus*, *Streptococcus pneumonia*) in Muller–Hinton agar (MHA) plates by the well diffusion method. Three replicates were performed for statistical analysis. The antibiotic discs (Chloramphenicol) were placed in the plate to test against each of the 6 organisms. About 5 µg and 10 µg of synthesised Ac-AgNPs and antibiotic discs were impregnated with the MHA medium. After 24 h period of incubation at 37 °C, the zone of inhibition was measured and recorded [37,38]. For statistical analysis, the software package of Graphpad Prism version 5, was used.

### 4.12. Antiplasmodial Activity

The filter-sterilised crude extract of Ac-AE, AgNO_3_ and Ac-AgNPs at various dosages (3.125, 6.25, 12.5, 25, 50 and 100 µg/mL) were added into the single-use 96-well plate, which was preloaded with two hundred microlitres of *P. falciparum* cultured in human red blood cells [39]. For the entire test sample, one negative control was maintained without adding the sample. For positive control, different concentrations of the standard drug, chloroquine, were also maintained [40]. The antiplasmodial effect of the positive control and the sample was estimated after 48 h of incubation by the Giemsa staining method [41]. The percentage of the antiplasmodial activity was measured by using the formula below:

Average % suppression of parasitaemia = [(Average % parasitaemia in control − Average % parasitaemia in test)/Average % parasitaemia in control] × 100

### 4.13. In Vitro Antidiabetic Activity

#### 4.13.1. α-Amylase Inhibitory Activity

The alpha amylase inhibitory activity of the plant sample and the synthesised silver nanoparticles was measured by adopting the standard protocol of Matsui et al. [42]. The amylase enzyme was procured from Himedia, Mumbai, India. The source of the enzyme is malt. Briefly, about 200 µL of the starch sample with a concentration of 0.4 mg/mL and one hundred microlitres of the samples with a concentrations of 20 mg/mL were added to 100 µL of the phosphate buffer. Instantly, 50 µL of the amylase solutions was incorporated into all the wells, and finally, the wells were made up to 500 µL volume using the solution of phosphate buffer. Then, the reaction mixtures were kept at room temperature for about 3 min to complete the chemical reaction. At the end, 1 mL of 0.1 N HCl was added to the entire well to terminate the reaction. Then, all the wells received 50 µL of iodine solution. Finally, the reaction was studied by taking the OD value of the sample mixtures at 660 nm using a spectrophotometer (Shimadzu 1601l; make: Shimadzu, Tokyo, Japan). The amount of alpha amylase inhibitory activity was calculated using the standard formula:

**Inhibition (%) = [(A_CB_ − A_c_) − (A_SB_ − A_S_)/A_CB_ − A_C_] × 100**

where **A_CB_** is the absorbance of the control blank, **A_C_** is the absorbance of the control, **A_SB_** is the absorbance of the sample blank, and **A_S_** is the absorbance of the sample.

#### 4.13.2. DPPH Radical Scavenging Assay

DPPH (1, 1-Diphenyl-2-picryl-hydrazyl) radical scavenging activities of samples and ascorbic acid were determined by adopting the protocol of Adeosun et al. [43]. In brief, 1 mL of 0.1 mM DPPH solution was combined with 2 mL of SW-AE, SW-AgNPs, and ascorbic acid (1 mg/mL) at various doses (3.125, 6.25, 12.50, 25, 50, and 100 g/mL). The reaction mixture was then agitated briefly and allowed to incubate for 30 min in complete darkness. At 517 nm, the OD values of the samples and the control were noted.

#### 4.13.3. Ferric Ion Reducing Power (FRAP) Assay

The FRAP scavenging ability of the samples and the standard was assessed using a slightly modified version of the established procedure [43]. About 20 mM/L ferric chloride hexahydrate, 10 mM/L TPTZ (2, 4, 6-tri [2-pyridyl]-s-triazine], and 300 mM/L acetate buffer (pH-3.6) solution were the ingredients in the manufacture of FRAP stock solution. By combining 4 mL of ferric chloride hexahydrate solution, 30 mL of acetate buffer, and 4 mL of TPTZ solution, the FRAP reagent’s working solution was prepared. Test tubes containing 2.5 mL of FRAP solution were filled with 500 micro litres (1 mg/mL) of various concentrations of the samples and ascorbic acid (3.125–100 g/mL), which were then incubated in the dark for 30 min. At 593 nm, the OD value of the sample and ascorbic acid was noted. For the tested assay, three replications were completed, and statistical analysis was performed.

#### 4.13.4. Hydrogen Peroxide Scavenging Assay

About 40 mM concentration of a hydrogen peroxide solution was prepared in a phosphate buffer with a pH of 7.4. Instantly, different concentrations of the samples and the standard (ascorbic acid) were added to the 96-well plates. The radical scavenging effects of the samples were assessed against 230 nm [44].

##### Statistic Evaluation

Using the following common formula, the samples’ in vitro antioxidant and diabetes enzyme inhibitory activities were determined.

**Inhibition (%) = [(A_0_ − A_1_)/A_0_] × 100**

where **A_0_** represents the control’s absorbance, and **A_1_** represents the sample’s absorbance. Using Graph Pad Prism version 5, the inhibitory concentration (IC_50_) values of the samples and standards were used to express their inhibitory activity (San Diego, CA, USA).

## 5. Conclusions

From the present study it has been concluded that Ac-AgNPs synthesised from the plant species of *Ageratum conyzoides* have been effectively proven to have antibacterial efficacy against the tested pathogens as well as antioxidant, antiplasmodial and antidiabetic activities. The major advantage of the present study is that the synthesised nanoparticles were optimised with different parameters, which can be an authentication for the results presented. We also suggest that the consumption of the plant part of *Ageratum conyzoides* may reduce the health issues generated by pathogenic microbes. Our future research warrants further in vivo and in silico studies of Ac-AgNPs against the important immunogenic proteins of the *Plasmodium falciparum* using molecular docking stimulations. Hence, the present report could form a baseline for effective antimicrobial formulations in the near future.

## Figures and Tables

**Table 1:** Phytochemical analysis of aqueous leaf extract of *Ageratum conyzoides* (AC-AE).

Phytochemical	Presence/Absence
Alkaloids	+
Flavonoids	−
Glycosides	−
Steroids	+
Terpenoids	−
Saponins	+
Tannins	+
Cardiac glycosides	+

+ Indicates presence; − indicates absence.

**Table 2:** Functional group analysis of the Ac-AE.

Frequency cm^−1^	Bond	Functional Group
3927.07	O-H Stretching	Alcohols, phenols
3842.20	O-H Stretching	Alcohol, phenols
3734.19	O-H Stretching	Alcohol, phenols
3383.14	N-H Stretching	Aliphatic primary amine
2792.93	C-H Stretching	Aldehyde
2337.72	O꞊C꞊O Stretching	Carbon dioxide
2106.27	C≡C Stretching	Allene
2005.97	N꞊C꞊S Stretching	Isothiocyanate
1928.82	C꞊C꞊C stretching	Allene
1643.35	C꞊N stretching	Imine/oxime
1531.48	N-O Stretching	Nitro compound
1377.17	O-H bonding	Alcohol
1253.73	C-O stretching	Aromatic ester
1215.15	C-O stretching	Vinyl ether

**Table 3:** Functional group analysis of the Ac-AgNPs.

Frequency cm^−1^	Bond	Functional Group
3873.06	O-H Stretching	Alcohols, phenols
3826.77	O-H Stretching	Alcohol, phenols
3387.00	C-H Stretching	Aldehyde
3348.42	O꞊C꞊O Stretching	Carbon dioxide
3271.27	C꞊N Stretching	Imine/oxime
2906.65	N-O Stretching	Nitro compound
2806.36	C-O stretching	Aromatic ester
2777.50	C-O stretching	Vinyl ether
2692.63	C꞊C꞊C stretching	Allene
2345.44	C꞊N stretching	Imine/oxime
2013.68	N꞊C꞊S Stretching	Isothiocyanate
1635.64	C꞊C꞊C stretching	Allene
1219.01	N꞊C꞊S Stretching	Isothiocyanate
1141.86	C꞊C꞊C stretching	Allene

**Table 4:** Percentage of suppression of parasitaemia after treatment with AC-AE, AC-AgNPs and AgNO_3_ at 24 h. Three replicates were performed.

	Percentage of Suppression of Parasitaemia at 24 h						
Samples/Conc.	100	50	25	12.5	6.25	3.125	IC_50_
**AC-AE**	58.22 ± 0.11	48.05 ± 0.05	40.01 ± 0.06	31.79 ± 0.14	24.58 ± 0.17	17.99 ± 0.07	68.03
**AC-AgNPs**	74.96 ± 0.05	69.64 ± 0.14	58.19 ± 0.15	49.90 ± 0.10	42.08 ± 0.07	38.99 ± 0.08	17.65
**AgNO_3_**	38.73 ± 0.14	29.08 ± 0.07	19.98 ± 0.07	12.66 ± 0.15	06.32 ± 0.15	0.00 ± 0.00	>100
**Chloroquine**	80.50 ± 0.23	72.97 ± 0.09	65.22 ± 0.13	49.85 ± 0.12	42.22 ± 0.08	30.48 ± 0.24	14.75
**Artemether**	82.21 ± 0.10	74.29 ± 0.18	68.24 ± 0.16	49.90 ± 0.08	43.17 ± 0.12	32.7 ± 0.21	17.65

**Table 5:** Percentage of inhibition of α-amylase after treatment with AC-AE, AC-AgNPs and AgNO_3_. Three replicates were performed.

Conc	Inhibition of α-Amylase			
	AC-AE	AC-AgNPs	AgNO_3_	Acarbose
**100**	78.63 ± 0.16	88.56 ± 0.14	50.79 ± 0.18	85.10 ± 0.10
**50**	65.49 ± 0.27	76.36 ± 0.14	40.54 ± 0.16	77.94 ± 0.09
**25**	50.70 ± 0.18	65.40 ± 0.27	35.57 ± 0.16	69.23 ± 0.18
**12.5**	41.39 ± 0.12	50.73 ± 0.17	20.70 ± 0.18	53.38 ± 0.15
**6.25**	30.30 ± 0.14	38.71 ± 0.16	12.79 ± 0.14	46.60 ± 0.18
**3.12**	19.54 ± 0.16	20.73 ± 0.13	5.38 ± 0.17	35.93 ± 0.12
**IC_50_**	**37.02**	**21.52**	**85.46**	**7.96**

## Data Availability

The data presented in this study are available on request from the corresponding authors.

## Supplementary Materials

The following supporting information [Supplementary Figures S1a–e and S2a–e] can be downloaded at: https://www.mdpi.com/article/10.3390/antibiotics12040688/s1 Supplementary Figure S1. UV spectra analysis of synthesized Ac-AgNPs at 1 mM (a) At pH-2 (b) At pH-4 (c) At pH-7 (d) At pH-8 and (e) At pH-10.; Supplementary Figure S2. UV spectra analysis of synthesized Ac-AgNPs at 5 mM. (a) At pH-2 (b) At pH-4 (c) At pH-7 (d) At pH-8 and (e) At pH-10.

## References

[1] WHO (2013). Diabetes. http://www.who.int/mediacentre/factsheets/fs312/en/.

[2] Abidullah S., Rauf A., Khan S.W., Ayaz A., Liaquat F., Saqib S. (2022). A comprehensive review on distribution; paharmacological uses and biological activities of *Argyrolobium roseum* (Cambess.) Jaub. & Spach. Acta Ecol. Sin..

[3] Bhatti H.A., Rubina R., Rashid F., Zaib S., Iqbal J., Hameed A. (2022). Synthesis and antitumor activities of novel Mannich base derivatives derived from natural flavonoids. Nat. Resour. Hum. Health.

[4] Margret Beula J., Sona Selva Malar S., Prasannakumar S., Ravikumar S., Kumaran R. (2015). Variable drug bio availability of native seaweeds in India for antimalarial therapeutics. Drug Discov..

[5] Sharma P., Mohan L., Srivastava C. (2009). *Amaranthus oleracea* and *Euphorbia hirta*: Natural potential larvicidal agents against the urban Indian malaria vector, *Anopheles stephensi* Liston (Diptera: Culicidae). Parasitol. Res..

[6] Sharma V.K., Yngard R.A., Lin Y. (2009). Silver nanoparticles: Green synthesis and their antimicrobial activities. Adv. Colloid Interface Sci..

[7] Klein G., Kim J., Himmeldirk K., Cao Y., Chen X. (2007). Antidiabetes and anti-obesity activity of *Lagerstroemia speciosa*. Evid. Based Complement. Altern. Med..

[8] Perez-Gutierrez R.M., Damian-Guzman M. (2012). Meliacinolin: A potent α-glucosidase and α-amylase inhibitor isolated from *Azadirachta indica* leaves and in vivo antidiabetic property in streptozotocin-nicotinamide-induced type 2 diabetes in mice. Biol. Pharm. Bull..

[9] Kotta J.C., Lestari A.B., Candrasari D.S., Hariono M. (2020). Medicinal effect, in silico bioactivity prediction, and pharmaceutical formulation of *Ageratum conyzoides* L.: A review. Scientifica.

[10] Shuaib M., Hussain F., Rauf A., Jan F., Romman M., Parvez R., Zeb A., Ali S., Abidullah S., Bahadur S. (2021). Traditional knowledge about medicinal plant in the remote areas of Wari Tehsil, Dir Upper, Pakistan. Braz. J. Biol..

[11] Das T.K., Ganguly S., Remanan S., Ghosh S., Das N.C. (2020). Mussel-inspired Ag/poly (norepinephrine)/MnO_2_ heterogeneous nanocatalyst for efficient reduction of 4-nitrophenol and 4-nitroaniline: An alternative approach. Res. Chem. Intermed..

[12] Das T.K., Ghosh S.K., Das N.C. (2023). Green synthesis of a reduced graphene oxide/silver nanoparticles-based catalyst for degradation of a wide range of organic pollutants. Nano-Struct. Nano-Objects.

[13] Chandraker S.K., Lal M., Ghosh M.K., Tiwari V., Ghorai T.K., Shukla R. (2020). Green synthesis of copper nanoparticles using leaf extract of *Ageratum houstonianum* Mill.; study of their photocatalytic and antibacterial activities. Nano Express.

[14] Madivoli E.S., Kareru P.G., Maina E.G., Nyabola A.O., Wanakai S.I., Nyang’au J.O. (2019). Biosynthesis of iron nanoparticles using *Ageratum conyzoides* extracts, their antimicrobial and photocatalytic activity. SN Appl. Sci..

[15] Wardani M., Yulizar Y., Abdullah I., Apriandanu D.O.B. (2009). Synthesis of NiO nanoparticles via green route using *Ageratum conyzoides* L. leaf extract and their catalytic activity. Proceedings of the IOP Conference Series: Materials Science and Engineering.

[16] Chandraker S.K., Lal M., Shukla R. (2019). DNA-binding, antioxidant, H_2_O_2_ sensing and photocatalytic properties of biogenic silver nanoparticles using *Ageratum conyzoides* L. leaf extract. RSC Adv..

[17] Puro K.N., Mazumder M.U., Khazeo P., Jyrwa R., Jamir N., Sailo L. Qualitative and quantitative analysis of phytochemicals of crude extracts of *Ageratum conyzoides* L. leaves. Proceedings of the Mizoram Science Congress 2018 (MSC 2018).

[18] Ahmed S., Ahmad M., Swami B.L., Ikram S. (2016). A review on plants extract mediated synthesis of silver nanoparticles for antimicrobial applications: A green expertise. J. Adv. Res..

[19] Rodríguez-Bernaldo de Quirós A., Frecha-Ferreiro S., Vidal-Pérez A.M., López-Hernández J. (2010). Antioxidant compounds in edible brown seaweeds. Eur. Food Res. Technol..

[20] Shittu K.O., Ihebunna O. (2017). Purification of simulated waste water using green synthesized silver nanoparticles of *Piliostigma thonningii* aqueous leave extract. Adv. Nat. Sci. Nanosci. Nanotechnol..

[21] Ojha S., Sett A., Bora U. (2017). Green synthesis of silver nanoparticles by *Ricinus communis* var. carmencita leaf extract and its antibacterial study. Adv. Nat. Sci. Nanosci. Nanotechnol..

[22] Patra N., Kar D., Pal A., Behera A. (2018). Antibacterial, anticancer, anti-diabetic and catalytic activity of bio-conjugated metal nanoparticles. Adv. Nat. Sci. Nanosci. Nanotechnol..

[23] Singh K., Naidoo Y., Mocktar C., Baijnath H. (2018). Biosynthesis of silver nanoparticles using *Plumbago auriculata* leaf and calyx extracts and evaluation of their antimicrobial activities. Adv. Nat. Sci. Nanosci. Nanotechnol..

[24] Bajpai V.K., Shukla S., Kang S.C. (2008). Chemical composition and antifungal activity of essential oil and various extract of *Silene armeria* L. Bioresour. Technol..

[25] Marshall K., Joint I., Callow M.E., Callow J.A. (2006). Effect of marine bacterial isolates on the growth and morphology of axenic plantlets of the green alga *Ulva linza*. Microb. Ecol..

[26] Duan X.J., Zhang W.W., Li X.M., Wang B.G. (2006). Evaluation of antioxidant property of extract and fractions obtained from a red alga, *Polysiphonia urceolata*. Food Chem..

[27] Sullivan R.T., Kim C.C., Fontana M.F., Feeney M.E., Jagannathan P., Boyle M.J., Drakeley C.J., Ssewanyana I., Nankya F., Mayanja-Kizza H. (2015). FCRL5 delineates functionally impaired memory B cells associated with *Plasmodium falciparum* exposure. PLoS Pathog..

[28] Grover J.K., Yadav S.P. (2004). Pharmacological actions and potential uses of *Momordica charantia*: A review. J. Ethnopharmacol..

[29] Ravikumar S., Jacob Inbaneson S., Suganthi P., Gokulakrishnan R., Venkatesan M. (2011). In vitro antiplasmodial activity of ethanolic extracts of seaweed macroalgae against *Plasmodium falciparum*. Parasitol. Res..

[30] Harborne A.J. (1998). Phytochemical Methods a Guide to Modern Techniques of Plant Analysis.

[31] Suresh J., Ragunath L., Hong S.I. (2019). Biosynthesis of mixed nanocrystalline Zn–Mg–Cu oxide nanocomposites and their antimicrobial behavior. Adv. Nat. Sci. Nanosci. Nanotechnol..

[32] Rao B., Tang R.C. (2017). Green synthesis of silver nanoparticles with antibacterial activities using aqueous *Eriobotrya japonica* leaf extract. Adv. Nat. Sci. Nanosci. Nanotechnol..

[33] SenthilKumar P., Sudha S. (2012). Evaluation of alpha-amylase and alpha-glucosidase inhibitory properties of selected seaweeds from Gulf of Mannar. Int. Res. J. Pharm..

[34] Anjum A., Shetty S.K.A., Ahmed M.A.N.Z.O.O.R., Sridhar B.K., Vijaya K.M.L. (2012). Development and validation of RPHPLC method for the quantitative estimation of cefadroxil monohydrate in bulk and Pharmaceutical dosage forms. Int. J. Chem. Sci..

[35] Moodley J.S., Krishna S.B.N., Pillay K., Govender P. (2018). Green synthesis of silver nanoparticles from *Moringa oleifera* leaf extracts and its antimicrobial potential. Adv. Nat. Sci. Nanosci. Nanotechnol..

[36] Deepak P., Amutha V., Birundha R., Sowmiya R., Kamaraj C., Balasubramanian V., Balasubramani G., Aiswarya D., Arul D., Perumal P. (2018). Facile green synthesis of nanoparticles from brown seaweed *Sargassum wightii* and its biological application potential. Adv. Nat. Sci. Nanosci. Nanotechnol..

[37] Oloninefa S.D., Moses Enemaduku A., Safiya Yahaya D., Abdullahi M. (2022). Characterization and Rate of Killing of Conjugated Silver Nanoparticles Against Selected Clinical Bacterial Isolates. Nat. Resour. Human Health..

[38] Saqib S., Faryad S., Afridi M.I., Arshad B., Younas M., Naeem M., Zaman W., Ullah F., Nisar M., Ali S. (2022). Bimetallic assembled silver nanoparticles impregnated in *Aspergillus fumigatus* extract damage the bacterial membrane surface and release cellular contents. Coatings.

[39] Rasoanaivo P., Petitjean A., Ratsimamanga-Urverg S., Rakoto-Ratsimamanga A. (1992). Medicinal plants used to treat malaria in Madagascar. J. Ethnopharmacol..

[40] Azas N., Laurencin N., Delmas F., Di Giorgio C., Gasquet M., Laget M., Timon-David P. (2002). Synergistic In vitro antimalarial activity of plant extracts used as traditional herbal remedies in Mali. Parasitol. Res..

[41] Waako P.J., Katuura E., Smith P., Folb P. (2007). East African medicinal plants as a source of lead compounds for the development of new antimalarial drugs. Afr. J. Ecol..

[42] Matsui T., Ueda T., Oki T., Sugita K., Terahara N., Matsumoto K. (2001). α-Glucosidase inhibitory action of natural acylated anthocyanins. 1. Survey of natural pigments with potent inhibitory activity. J. Agric. Food Chem..

[43] Adeosun A.M., Oni S.O., Ighodaro O.M., Durosinlorun O.H., Oyedele O.M. (2016). Phytochemical, minerals and free radical scavenging profiles of *Phoenix dactilyfera* L. seed extract. J. Taibah Univ. Med. Sci..

[44] Al-Amiery A.A., Al-Majedy Y.K., Kadhum A.A.H., Mohamad A.B. (2015). Hydrogen peroxide scavenging activity of novel coumarins synthesized using different approaches. PLoS ONE.

