# Supplementary material for: One-Pot Synthesis of Silver Nanoparticles Derived from Aqueous Leaf Extract of Ageratum conyzoides and Their Biological Efficacy
Source: Antibiotics (Basel). 2023 Apr 1;12(4):688. doi: 10.3390/antibiotics12040688 (PMC10135330; doi:10.3390/antibiotics12040688)
Supplement: Supplementary file 1 [file antibiotics-12-00688-s001.zip › antibiotics-2310773-supplementary.pdf]

Supplementary File

a

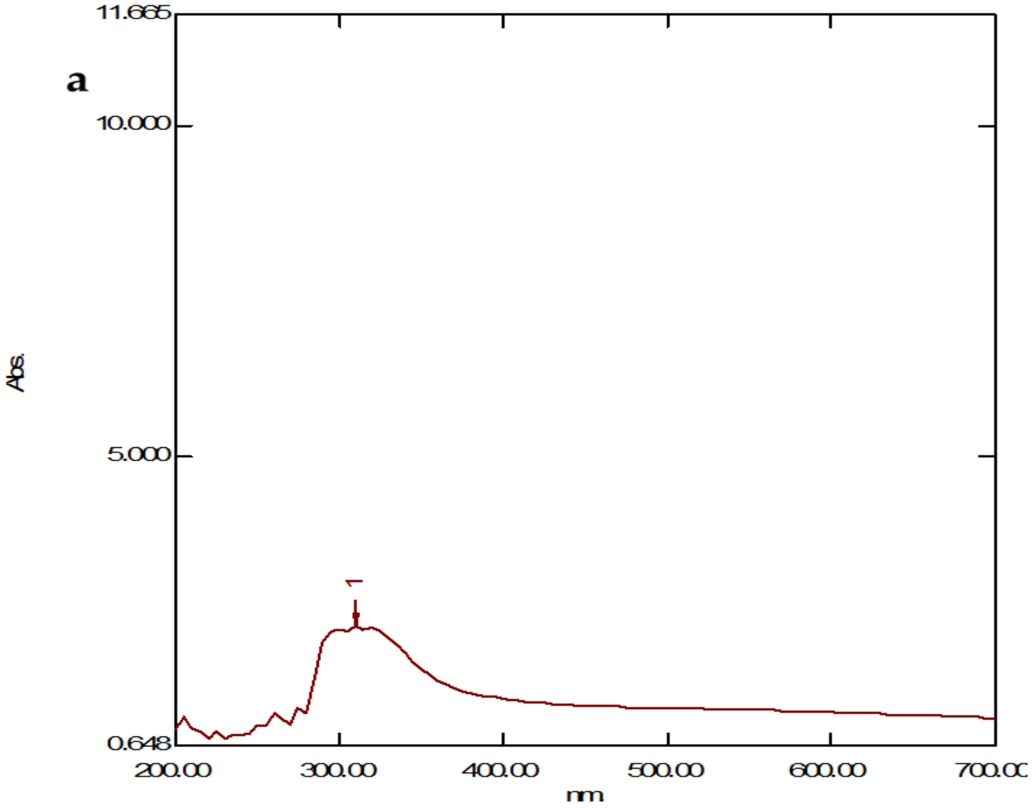

b

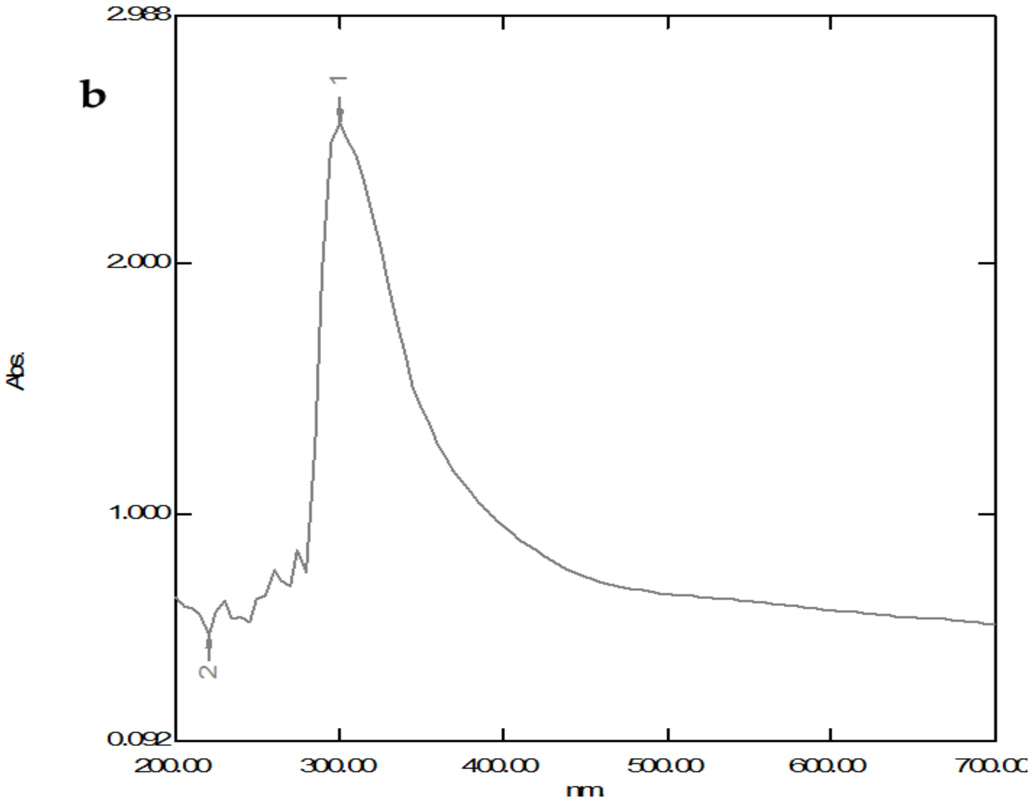

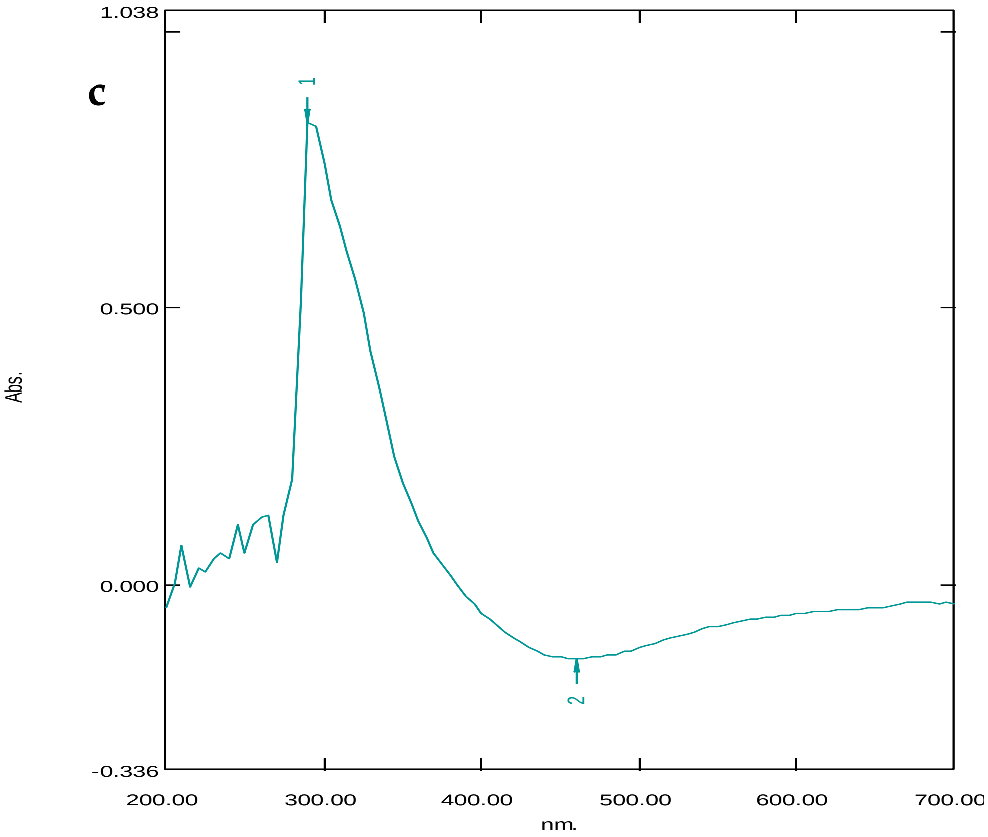
c
1.038
0.500
0.000
-0.336
Abs.
200.00
300.00
400.00
500.00
600.00
700.00
nm.
1
2

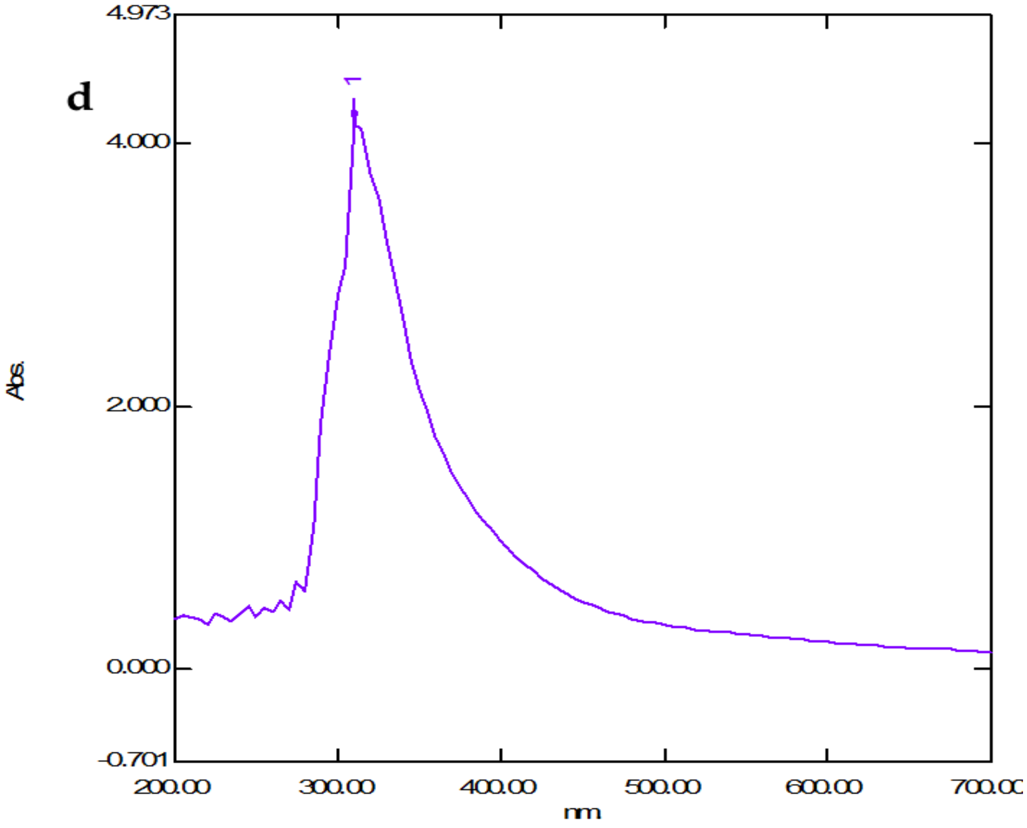
d
4.973
4.000
2.000
0.000
-0.701
Abs.
200.00
300.00
400.00
500.00
600.00
nm
1

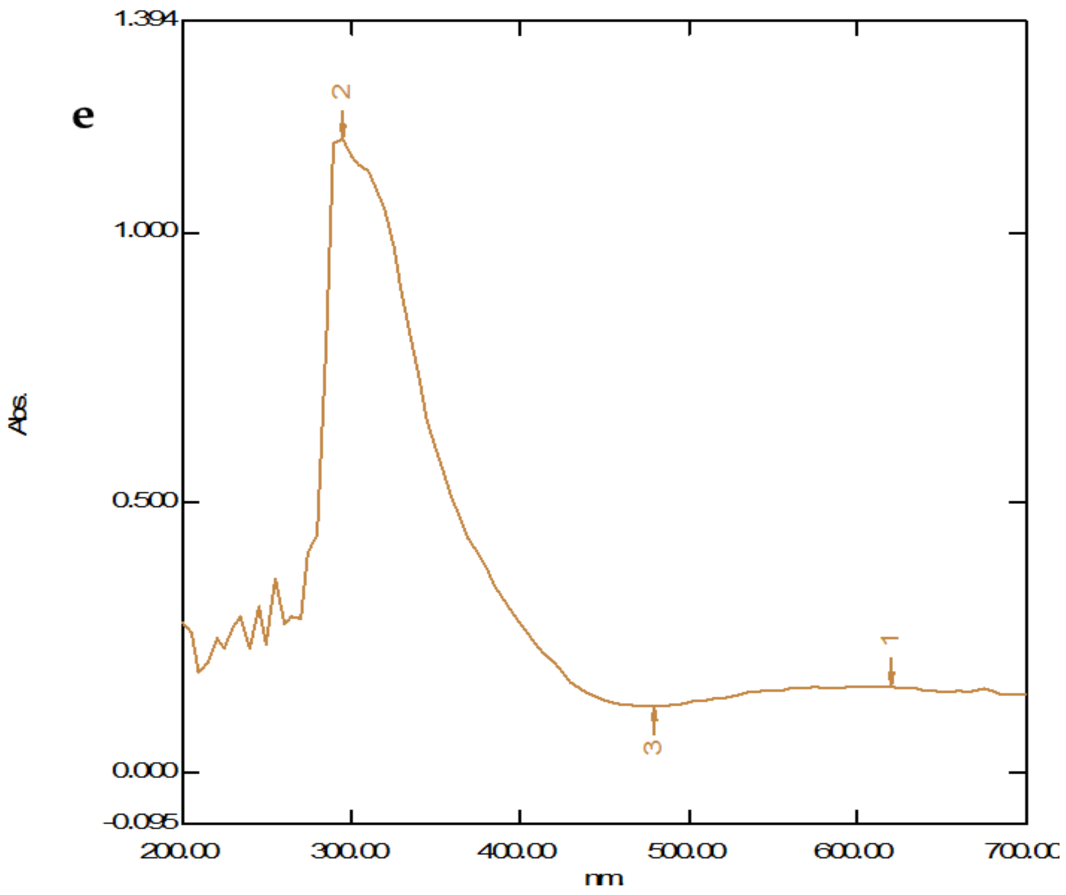

Figure S1. UV spectra analysis of synthesized Ac-AgNPs at 1 mM a) At pH-2 b) At pH-4 c) At pH-7 d) At pH-8 and e) At pH-10.

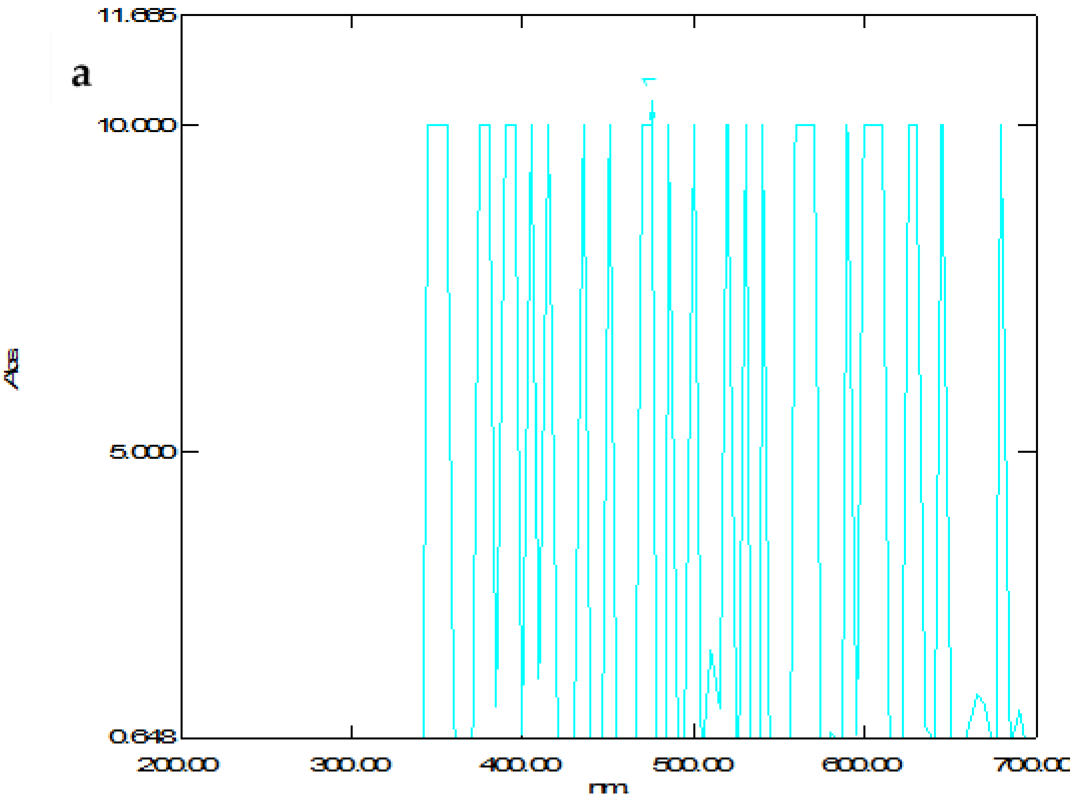
a
11.665
10.000
5.000
0.648
Abs
200.00
300.00
400.00
500.00
600.00
700.00
nm

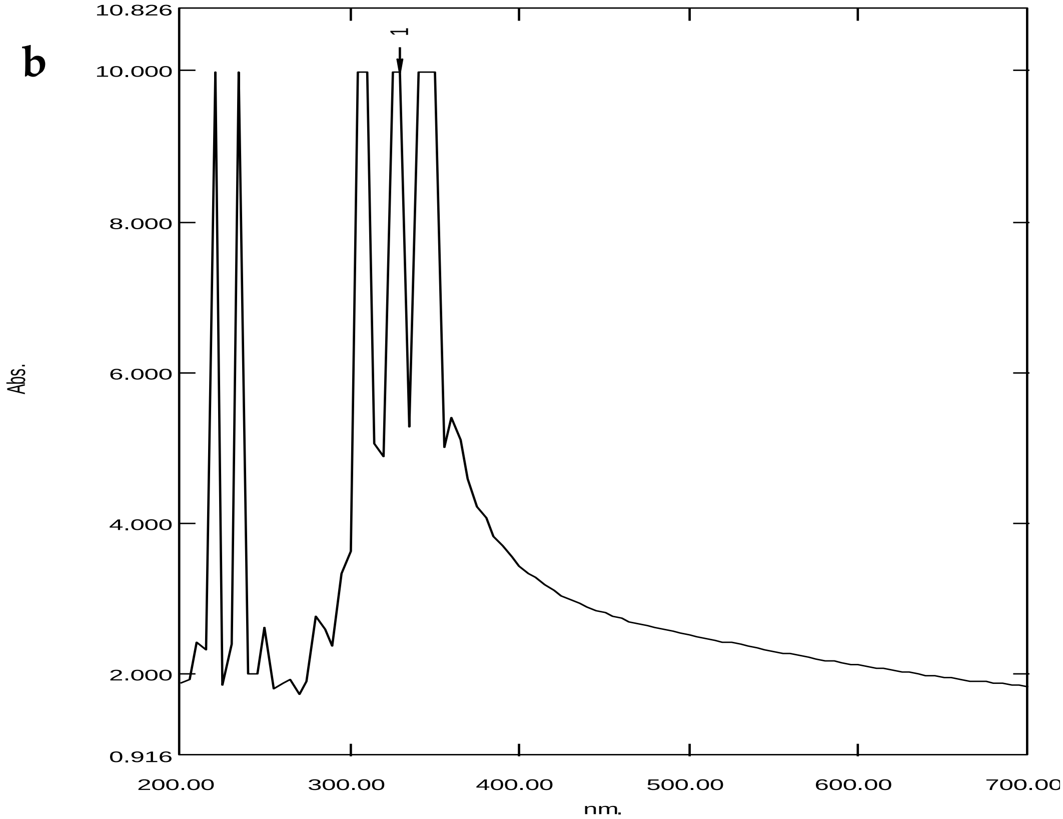
b
10.826
10.000
8.000
6.000
4.000
2.000
0.916
Abs.
1
200.00
300.00
400.00
500.00
600.00
700.00
nm.

c

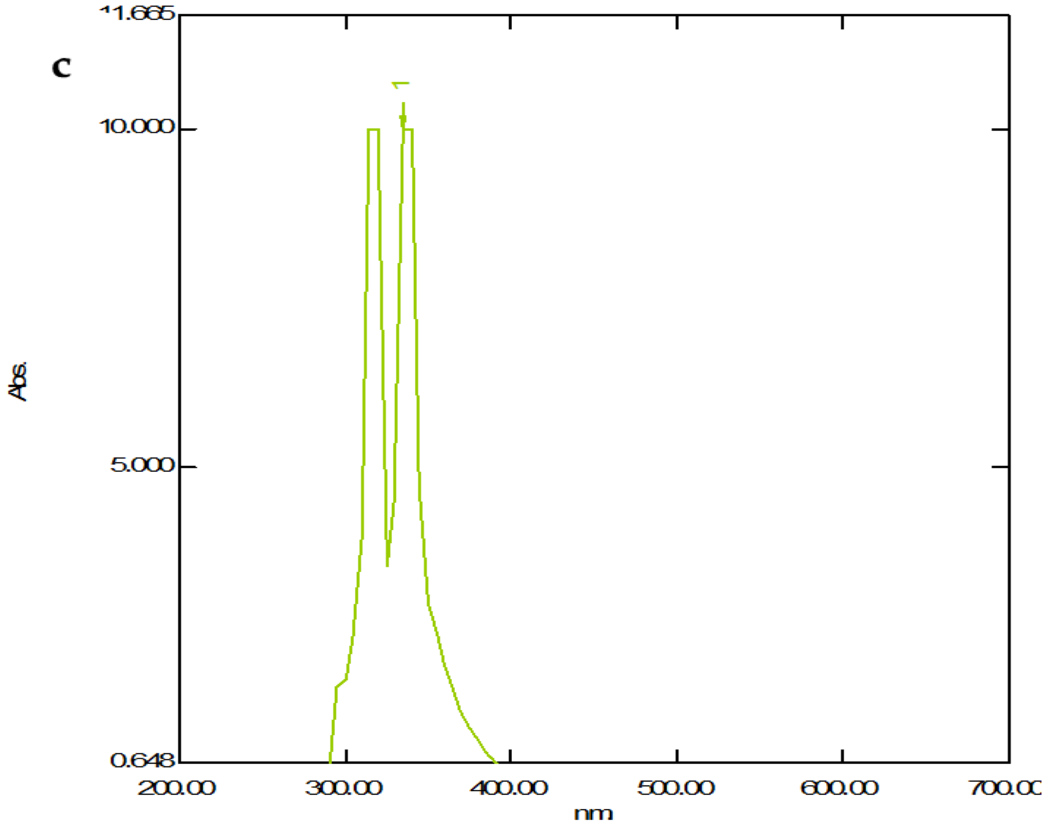
11.665
10.000
5.000
0.648
Abs.
200.00
300.00
400.00
500.00
600.00
700.00
nm

d

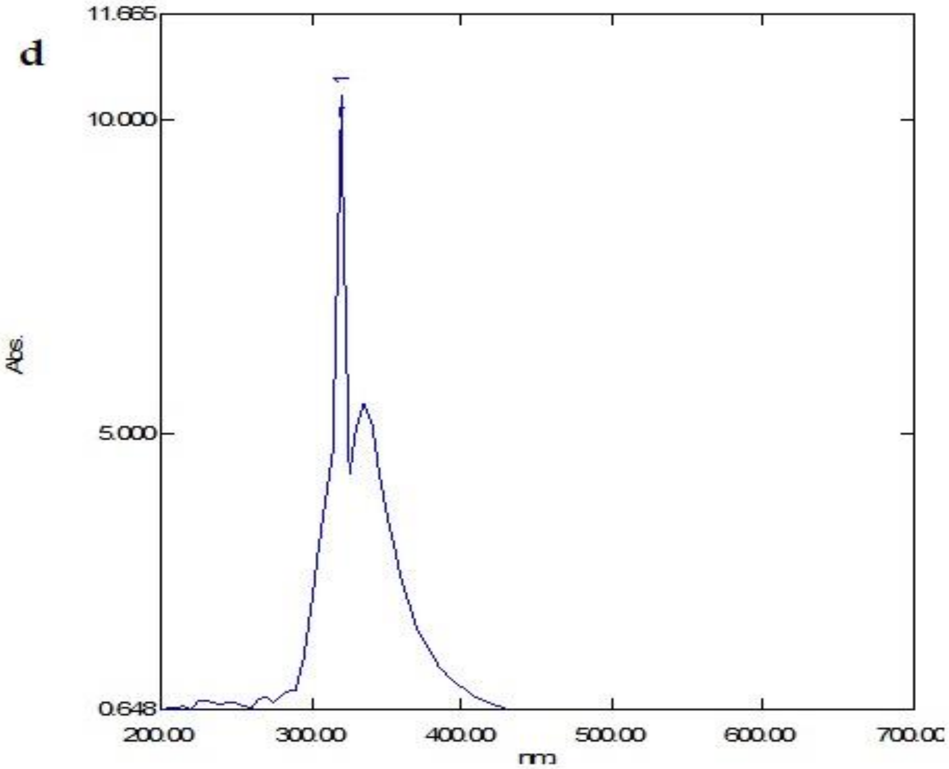
11.665
10.000
5.000
0.648
Abs.
200.00
300.00
400.00
500.00
600.00
nm

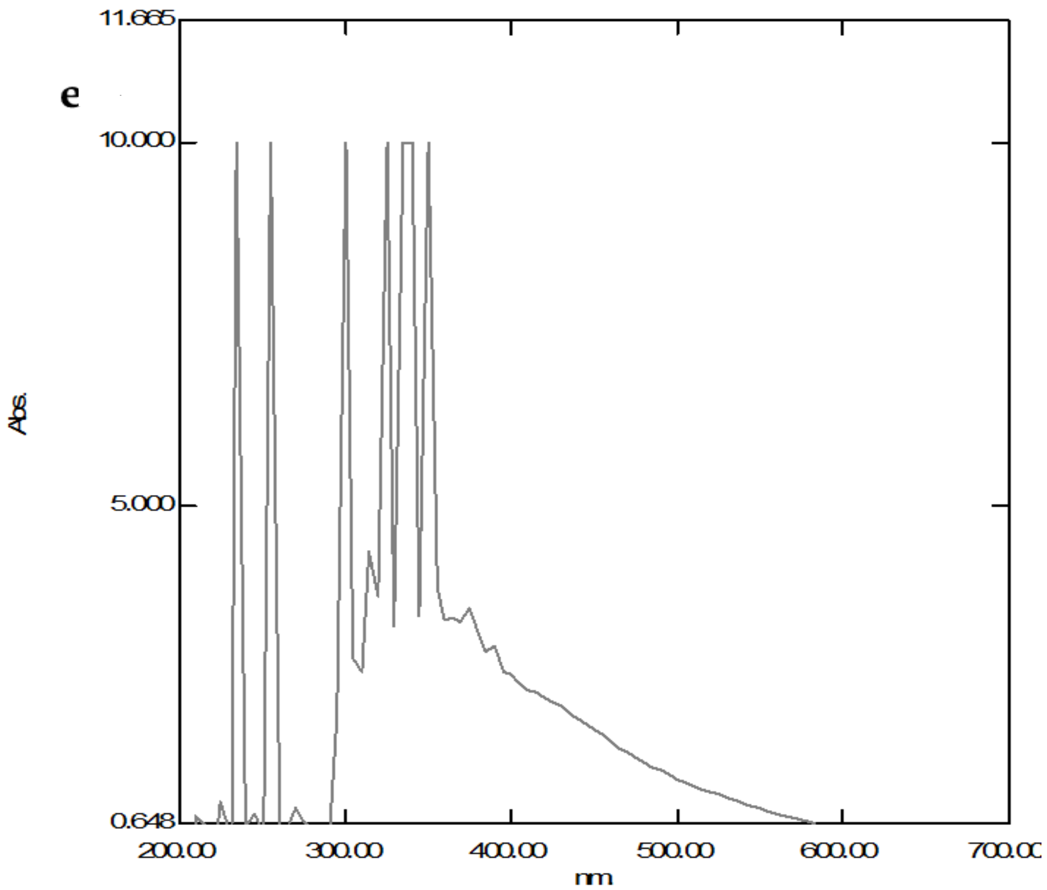

Figure S2. UV spectra analysis of synthesized Ac-AgNPs at 5 mM. a) At pH-2 b) At pH-4 c) At pH-7 d) At pH-8 and e) At pH-10.
